# A Non-Classical LysR-Type Transcriptional Regulator PA2206 Is Required for an Effective Oxidative Stress Response in *Pseudomonas aeruginosa*

**DOI:** 10.1371/journal.pone.0054479

**Published:** 2013-01-28

**Authors:** F. Jerry Reen, Jill M. Haynes, Marlies J. Mooij, Fergal O'Gara

**Affiliations:** BIOMERIT Research Centre, Department of Microbiology, University College Cork, Cork, Ireland; East Carolina University School of Medicine, United States of America

## Abstract

LysR-type transcriptional regulators (LTTRs) are emerging as key circuit components in regulating microbial stress responses and are implicated in modulating oxidative stress in the human opportunistic pathogen *Pseudomonas aeruginosa*. The oxidative stress response encapsulates several strategies to overcome the deleterious effects of reactive oxygen species. However, many of the regulatory components and associated molecular mechanisms underpinning this key adaptive response remain to be characterised. Comparative analysis of publically available transcriptomic datasets led to the identification of a novel LTTR, PA2206, whose expression was altered in response to a range of host signals in addition to oxidative stress. PA2206 was found to be required for tolerance to H_2_O_2_ *in vitro* and lethality *in vivo* in the Zebrafish embryo model of infection. Transcriptomic analysis in the presence of H_2_O_2_ showed that PA2206 altered the expression of 58 genes, including a large repertoire of oxidative stress and iron responsive genes, independent of the master regulator of oxidative stress, OxyR. Contrary to the classic mechanism of LysR regulation, PA2206 did not autoregulate its own expression and did not influence expression of adjacent or divergently transcribed genes. The *PA2214-15* operon was identified as a direct target of PA2206 with truncated promoter fragments revealing binding to the 5′-ATTGCCTGGGGTTAT-3′ LysR box adjacent to the predicted −35 region. PA2206 also interacted with the *pvdS* promoter suggesting a global dimension to the PA2206 regulon, and suggests PA2206 is an important regulatory component of *P. aeruginosa* adaptation during oxidative stress.

## Introduction

Bacteria have evolved diverse integrated regulatory networks which are essential for their survival and persistence in a vast array of ecological niches. An efficient response to changing environmental conditions requires the coordination of signal perception and transduction, transcriptional and post-transcriptional regulation, as well as post-translational events. By far the largest known family of transcriptional regulators in prokaryotes, LysR-type transcriptional regulators (LTTRs), are emerging as key coordinators of microbial gene expression. LTTRs are ubiquitous among bacteria with orthologues also found in archaea and eukaryotic organisms [1]. These signal responsive proteins represent the primary mechanism of regulation of catabolic systems in bacteria while they have also been shown to influence key adaptive and virulence phenotypes such as biofilm formation, motility, signalling, secondary metabolite production, and oxidative stress caused by reactive oxygen species (ROS) [1], [2].

Organisms are exposed to ROS, such as hydrogen peroxide (H_2_O_2_) and the superoxide anion, during the course of normal aerobic metabolism or following exposure to radical-generating compounds, including redox-cycling drugs. ROS cause wide-ranging damage to macromolecules, which can eventually lead to cell death. To protect themselves against this damage, cells have evolved effective defence mechanisms, including anti-oxidant enzymes and free radical scavengers. OxyR, an LTTR that is universally encoded among Gram negative bacteria, is considered the master regulator of the oxidative stress response. In *Escherichia coli*, OxyR works in concert with the small RNA *oxyS* to coordinate expression of catalase and peroxidase genes [3]. More recently, several other LTTRs have been shown to play a role in the oxidative stress response, including Hrg in *Salmonella enterica* [4], HypR in *Enterococcus faecalis* [5], CztR in *Caulobacter crescentus* [6] and HcaR in *E. coli* [7]. However, with the exception of OxyR, the role of LTTRs during oxidative stress remains largely uncharacterised in many bacteria. This is particularly true for the important nosocomial pathogen *Pseudomonas aeruginosa* in which LTTRs account for more than 2% of the total gene content [8].

In addition to being a leading cause of hospital-acquired infections in immunocompromised individuals, *P. aeruginosa* is also the primary cause of chronic lung infections in patients suffering from cystic fibrosis [9], [10], [11], [12], [13]. OxyR has been shown to modulate the expression of several key oxidative stress response genes including *katA*, *katB*, *ahpB* and *ahpCF* in *P. aeruginosa* [14], [15]. Recently, the OxyR regulon in *P. aeruginosa* was defined through Co-IP analysis and was shown to include genes involved in iron homeostasis, quorum sensing, protein synthesis, and oxidative phosphorylation [15]. Wei and colleagues proposed a multilayered response to oxidative stress which includes increased expression of antioxidants to detoxify ROS, inhibition of primary metabolism and oxidative respiration to reduce the production of ROS, and modulation of iron uptake systems to minimise the Fenton reaction [15]. This requires a complex cellular response, the control of which is likely to encompass multiple regulatory components. However, apart from OxyR, the role of LTTR proteins in the *P. aeruginosa* oxidative stress response has not been investigated.

In this study, we identified PA2206 as a novel LTTR transcriptionally influenced by both oxidative stress and host signals, independent of the master regulator of oxidative stress, OxyR. PA2206 was found to be required for an effective oxidative stress response as well as lethality in a zebrafish model of infection. This novel non-classical LTTR was found to have a significant influence on the expression of oxidative stress responsive genes, while the *PA2214-2215* and *pvdS* promoters were identified as direct targets. PA2206 was shown to interact specifically with the 5′-ATTGCCTGGGGTTAT-3′ LysR box adjacent to the predicted −35 region of the *PA2214-15* promoter. The requirement of PA2206 for tolerance to oxidative stress and zebrafish embryo lethality, independent of OxyR, implies a novel route for the management of oxidative stress in *P. aeruginosa*, and a role for PA2206 during the host-microbial interaction.

## Materials and Methods

### Bacterial strains, media and growth conditions

Strains and plasmids used in this study are listed in Table 1. All cultures of *P. aeruginosa* strain mPAO1 and associated mutants were routinely grown in Luria Bertani [16] or M9 minimal media [supplemented with 0.3% (w/v) sodium citrate, 1 mM MgSO_4_, and 0.05 mM FeCl_3_] at 37°C [17]. Bacterial cultures were grown in Casamino Acid (CAA) medium supplemented with 100 µM FeCl_3_, for the proteomic analysis [18]. Tetracycline-resistant transposon insertion mutants (Table 1) were obtained from the University of Washington Genome Centre mutant library [19]. Transposon insertion, location, and orientation were confirmed for all mutants. *E. coli* strains were routinely grown in LB media at 37°C. Where appropriate, antibiotics were added to growth media at the following concentrations: tetracycline, 60 µg ml^−1^, and gentamicin, 20 µg ml^−1^, for *P. aeruginosa*; and ampicillin, 50 µg ml^−1^, and gentamicin, 20 µg ml^−1^, for *E. coli*.

**Table 1:** Bacterial strains and plasmids used in this study.

Strains	Description	Source/Reference
***P. aeruginosa***		
mPAO1	Wild-type	[19]
*mPA2206^−^*	*mPA2206::Tn5* Tc^R^	[19]
*mPA2206^C^*	*PA2206^−^* containing pBR-*PA2206^P^*	This study
*mPA2215*	*mPA2206::Tn5* Tc^R^	[19]
*oxyR^−^*	PAO1 *oxyR^−^*	[14]
***E. coli***		
TOP 10	F– mcrA Δ(mrr-hsdRMS-mcrBC) Φ80lacZΔM15 ΔlacX74 recA1 araD139 Δ(ara leu) 7697 galU galK rpsL (StrR) endA1 nupG	Invitrogen
DH5α	F– Φ80*lac*ZΔM15 Δ(*lac*ZYA-*arg*F) U169 *rec*A1 *end*A1 *hsd*R17 (rK−, mK+) phoA supE44 λ– thi-1 gyrA96 relA1	Invitrogen
BL21-CodonPlus (DE3)	*E. coli* B F- *ompT* hsdS(rB− mB−) *dcm+* Tetr *E. coli* gal λ (DE3) endA Hte [argU ileY leuW Camr]	Merck
EcpBR	*E. coli* DH5α containing pBBR1MCS5 plasmid.	This study
EcpBR-2	*E. coli* DH5α containing *pBR-2206*.	This study
**Plasmids**		
pCR2.1-TOPO TA	Cloning vector, Ap^r^, Km^R^	Invitrogen
pMP190	IncQ origin, low-copy-number *lacZ* fusion vector; Cm^r^ Str^r^	[27]
pMP-p2214	pMP190-derived PA2214 promoter-*lacZ* fusion plasmid	This study
pMP-p2216	pMP190-derived PA2216 promoter-*lacZ* fusion plasmid	This study
pMP-p2206	pMP190-derived PA2206 promoter-*lacZ* fusion plasmid	This study
pET28a	T7 promoter-driven His-tag protein expression vector, Km^r^	Novagen
pBBR1MCS5	Broad host range complementation plasmid, *lacZ alpha*, Gm^r^	[26]
pBR-*2206^P^*	pBBR1MCS5 containing PAO1 *PA2206* with native promoter downstream of lac promoter	This study
pBR-*2206*	pBBR1MCS5 containing PAO1 *PA2206* CDS downstream of lac promoter	This study

### Assay of survival under conditions of oxidative stress

Growth assays were performed with 20 mM H_2_O_2_ or 40 mM menadione added to exponentially growing cells. Briefly, overnight cultures were transferred into fresh media at a starting OD600 nm of 0.1, and were incubated at 37°C with shaking for 3 hrs. Subsequently, at approximately OD600 nm 0.6, H_2_O_2_ or menadione was added and growth monitored hourly. Survival assays were performed in the presence of 100 mM H_2_O_2_ as this concentration has previously been chosen for *P. aeruginosa* and other species [20], [21]. Overnight cultures were washed in Ringer's solution (¼-strength) and resuspended to an optical density of 0.8 at 600 nm. Bacterial suspensions (5 ml) were exposed to 100 mM H_2_O_2_ at 37°C with agitation (150 rpm). Viable counts were determined on LB agar with antibiotic selection where appropriate before and after 1 hour of exposure, and expressed as percentage surviving cells as described by Heeb and colleagues [22]. Zonal inhibition assays were performed on M9 plates supplemented with glucose (2% w/v). An overnight PAO1 culture was spread on the agar plate using a sterile swab and air dried in a laminar flow hood for 30 mins. Subsequently, filter paper disks (approx 7 mm) were placed in the centre of the plate and saturated with 10 µl of a 30% (w/w) solution of H_2_O_2_ (Sigma) corresponding to an 8.8 M concentration. Plates were incubated at 37°C overnight and the zone of inhibition measured at 16 hrs.

### Zebrafish infection assay

Zebrafish embryos were derived from adults of the Zebrafish AB line (*Danio rerio*) which were kept at 28°C in a 14-h/10-h light/dark cycle in our breeding facility. Zebrafish embryos were staged at 26 hours post fertilization according to previously described developmental criteria [23]. Embryos were manually dechorionated and subsequently anaesthetized in a 0.015% aqueous solution of ethyl-3-aminobenzoate methanesulfonate salt. *P. aeruginosa* cells from an overnight culture in LB broth were washed twice in phosphate buffer saline and diluted in phenol red phosphate buffer saline in order to visually monitor injection under a stereo-microscope. Bacterial cells were then micro-injected (1–2 nl) into the blood island of the Zebrafish embryos. The inoculum size was determined by injecting in triplicate in phosphate buffer saline followed by plating on LB agar plates. Approximately 300 colony forming units were injected into 10 embryos per group. Injected Zebrafish embryos were returned to egg water, incubated at 30°C, and monitored for mortality on a daily basis. Data represents three independent biological replicates. Kaplan-Meier curves were used to compare survival rates of zebrafish infected with wild-type and *PA2206* ^−^. Differences between infected embryos, compared using a log-rank test in MedCalc®, were considered significant ≤0.05. All animal experiment protocols were approved by the Animal Experimentation Ethics Committee, University College Cork.

### Bioinformatics and comparative genomic analysis

Genome sequences were obtained from the www.pseudomonas.com resource [24] and compared using the ARTEMIS Comparison Tool on the www.webact.org site [25]. Promoter predictions were performed using BProm software on the http://linux1.softberry.com site.

To screen the PAO1 genome for the LysR consensus sequences identified proximal to the *PA2214* predicted −35 upstream region, nucleotide sequences from positions −300 to −1 relative to the origin of translation of all coding sequences (CDS) were parsed from the Genbank annotated genome sequence file (version AE004091.2) of *P. aeruginosa* PAO1 and stored locally in FASTA format using a script in Perl. CDSs matching TTN_9_TA, where N is any nucleotide, in the 300 bp upstream of the origin of translations were identified using Bioperl [16]. Subsequently, sequences were analysed using MEME motif search software to construct a PA2206 LysR consensus motif. This analysis was restricted to sequences which had a statistical significance (p-value) lower than 1e^−04^.

### Complementation *in trans* of mutant strains

Oligonucleotide primers PA2206CCF and PA2206CCR (Table S1) were designed to amplify the *PA2206* gene and promoter region based on the PAO1 genome sequence (GenBank accession number NC002516). The DNA fragment was amplified from PAO1 genomic DNA using Platinum High Fidelity Taq polymerase (Invitrogen, UK) and subsequently cloned as a *Hind*III and *Xba*I fragment into *Hind*III-*Xba*I sites of pBBR1MCS-5 generating pBR1-*PA2206* ^C^ [26]. Similarly, primer Hin2206F was designed at the *PA2206* translational start site to create construct (pBR1-2) with primer Xb2206R (Table S1). Chemical transformation into competent *E. coli* DH5α cells and purification was followed by electroporation into the *P. aeruginosa* mPAO1 *PA2206* Tn5 mutant strain as described by Sambrook and colleagues [17]. Construct fidelity was confirmed by sequencing in both directions.

### Promoter-fusion analysis

Promoter regions were amplified from PAO1 genomic DNA by PCR using Platinum High Fidelity Taq polymerase (Invitrogen). The *PA2214* promoter region was amplified using PA2214TRF and PA2214TRR primers, while the *PA2216* promoter was amplified using PA2216TRF and PA2216TRR (Table S1). The *PA2206* promoter was amplified using PA2206TRF and PA2206TRR primers (Table S1). Inserts were digested using *Xba*I*-Kpn*I and subsequently ligated directly into similarly digested pMP190 [27] plasmid at 16°C overnight followed by transformation into chemically competent DH5α *E. coli* cells. Promoter orientation was confirmed by PCR using a promoter specific primer and a *lacZ* primer and the fidelity of all transcriptional fusions was verified by sequencing (GATC). Triparental mating utilising an *E. coli* strain harbouring the pRK2013 helper plasmid was performed to transfer the fusions into recipient *P. aeruginosa* strains. The *PA2214* promoter fusion was also introduced into *E. coli* containing pBR1-2 and pBBR1MCS5 vector control. Fusion analysis was performed in M9 media supplemented with glucose (10 mM) with cultures grown at 37°C with shaking.

### Microarray analysis

Three independent overnight cultures of *P. aeruginosa PA2206^−^* mutant and *PA2206^C^* grown in M9 minimal media [supplemented with 1 mM MgsO_4_ and 2% glucose] with shaking at 37°C, were diluted to an optical density of 0.05 at 600 nm in fresh media. Cells were then grown to an optical density of 0.5 at 600 nm at which stage they were exposed to 1 mM H_2_O_2_ for a ten minute time period. Total RNA was subsequently extracted using the QIAGEN™ RNeasy^®^ Mini RNA extraction kit in accordance with the manufacturer's guidelines. RNA was treated with Ambion^®^ TURBO™ DNase at 37°C for 1 hour and the RNA pellet was then resuspended in sterile diethylpyrocarbonate (DEPC)-treated water (1 ml of 0.1% DEPC) to a final volume of 25 µl. Isolated RNA was sent to DNAVision (Belgium) for further analysis. RNA quality was checked using a Bioanalyzer Agilent 2100 at DNAVision, and cDNA synthesis, fragmentation and terminal labelling preceded hybridisation on Affymetrix GeneChip *P. aeruginosa* Genome arrays according to the Affymetrix guidelines. The raw data was normalised using the robust multiarray average (RMA) algorithm [28] and pairwise comparison (t-test, P<0.05) was carried out to generate a list of genes with significantly altered expression between the test strains of greater than 1.5 fold. The p-value was then assessed for each gene by controlling the false discovery rate using the Benjamini Hochberg method [29]. All files have been submitted to the GEO database (Ref No: GSE23367) compliant with MIAME.

### Real-time quantitative RT-PCR

cDNA was synthesised using AMV Reverse Transcriptase (Promega), RNasin (100 U/µl) (Promega), random primers (0.5 µg/µl) (Promega) and 10 mM dNTPs (Promega). Specific RT-PCR primers were designed for each gene based on the PAO1 genome sequence (Table S1). Standards were generated by PCR and quantitative real-time PCR analysis of expression was carried out using the Quantifast SYBR Green PCR kit (QIAGEN). Quantitative real time-PCR signals were normalised to the constitutively expressed housekeeping gene *proC* [30], whose expression was shown to be unaltered under the conditions used to generate the transcriptomic profile.

Microarray data were validated by quantitative RT-PCR analysis of RNA isolated from three independent experiments as above. For further analysis of gene expression under different oxidative stress conditions, overnight cultures of the wild-type and mutant strains were inoculated into M9 minimal media supplemented with glucose (2% w/v) at an initial OD_600 nm_ of 0.05. The cells were grown to an OD_600 nm_ of 0.4 and the cultures were subsequently divided and exposed to 1 mM and/or 5 mM concentrations of H_2_O_2_ for 5 and 10 minute time periods. Untreated cells were harvested as a control against which to compare gene expression. All assays were carried out in triplicate, and the mean and standard error were calculated.

### PA2206-HisTag protein purification and quantification

The *PA2206* gene from *P. aeruginosa* PAO1 was amplified using gene-specific primers 2206HisF and 2206HisNR (Table S1) and cloned into the pET28a inducible expression plasmid (Invitrogen) using the *Eco*RI-*Hind*III restriction enzymes (New England Biolabs). Constructs were transformed into *E. coli* BL21 Codon Plus (DE3) cells giving rise to *PA2206* fused to an N-terminal His_6_ tag. Overnight LB cultures were transferred into fresh media supplemented with appropriate antibiotics and were grown at 30°C to an optical density of 0.8 at 600 nm at which time IPTG was added to a final concentration of 0.1 mM. The cultures were grown for a further 4 hrs and cells were harvested at 4°C following centrifugation at 10,000 rpm for 10 mins. Supernatants were removed and cells were stored overnight a −80°C to enhance lysis. Cell pellets were resuspended in Cell Lytic™ B, lysosyme, benzonase, and protease inhibitors (all Sigma-Aldrich), and subsequently incubated at room temperature for 15 mins with shaking (100 rpm). Following centrifugation at 10,000 rpm at 4°C, the clarified supernatant was purified on a Poly-Prep chromatography column (Bio-Rad) containing His Select nickel affinity gel (Sigma-Aldrich) and eluted using increasing concentrations of imidazole (10–500 mM). Fractions were analysed on a 10% (w/v) SDS PAGE (10%) gels (Fig. S1), and the concentration of purified PA2206-His_6_ protein was determined by Bradford assay as described before [31].

### Electrophoretic mobility shift analysis (EMSA)

Target promoter DNA was amplified using InfraRed end-labelled promoter-specific oligonucleotides (Table S1). Promoter fragments were purified using the QIAGEN gel extraction kit and the InfraRed signal was verified on 6% polyacrylamide gels using the Odyssey Imager (LI-COR Biosciences). Truncated promoter fragments were amplified containing boxes 1 & 2 (PA2214F2 and PA2214 TrR), boxes 3–8 (PA2214TrF and PA2214R), boxes 4–8 (PA2214TrAF and PA2214TrAR), boxes 5–8 (PA2214TrBF and PA2214TrAR), boxes 6–8 (PA2214TrCF and PA2214TrAR), boxes 7–8 (PA2214TrDF and PA2214 TrER) and box 8 (PA2214TrEF and PA2214 TrER), respectively (Table S1). Protein-DNA binding assays containing 15 fmoles of DNA with increasing concentrations of PA2206 protein were performed at room-temperature for 30 mins in Roche 5× binding buffer (Roche, Ireland). After completion of the binding assay, 4 µl of 50% glycerol/orange G buffer was added to the samples (20 µl), which were subsequently analysed on a 6% polyacrylamide gel in 0.5× TBE buffer.

## Results

### Comparative transcriptomic analysis reveals a non-classical host-responsive LysR-Type Transcriptional Regulator (LTTR)

The oxidative stress response is central to the survival of many microbial pathogens during infection. However, the molecular mechanisms governing this key adaptive phenotype remain largely uncharacterised. Interrogation of the publically available transcriptome datasets revealed a striking correlation between oxidative stress and the induction of genes encoding LTTRs in *P. aeruginosa*. Expression of 41 out of 121 genes annotated as LTTRs in the PAO1 genome was ≥2-fold altered (39 upregulated) at the transcriptional level in *P. aeruginosa* in response to oxidative stress induced by H_2_O_2_ [32], [33], [34]. However, surprisingly only a limited number of these LTTRs that respond to oxidative stress were also altered in transcriptomic datasets designed to investigate the host interaction [35], [36], [37]. We considered this subset of LTTRs likely to play an important role in *P. aeruginosa* host-microbial interactions and selected PA2206 on the basis of it being regulated in response to both human (+2.82 fold) and plant (+2.7 fold) signals, in addition to oxidative stress [33], [35], [37]. Annotated as a PcaQ-type LTTR and a unique transcriptional unit, PA2206 was not found to be divergently transcribed with an adjacent gene, in contrast to the classic LysR topology [1]. Furthermore, quantitative RT-PCR and promoter fusion analysis revealed that PA2206 did not influence the expression of adjacently transcribed *PA2205* or *PA2207-09* (data not shown). Therefore, PA2206 would appear to be one of an emerging class of non-classical LTTRs, and its induction in the presence of a range of host signals suggests an important role during the host-interaction.

### Expression of *PA2206* is induced in response to hydrogen peroxide and menadione, independent of OxyR

**Figure 1:**
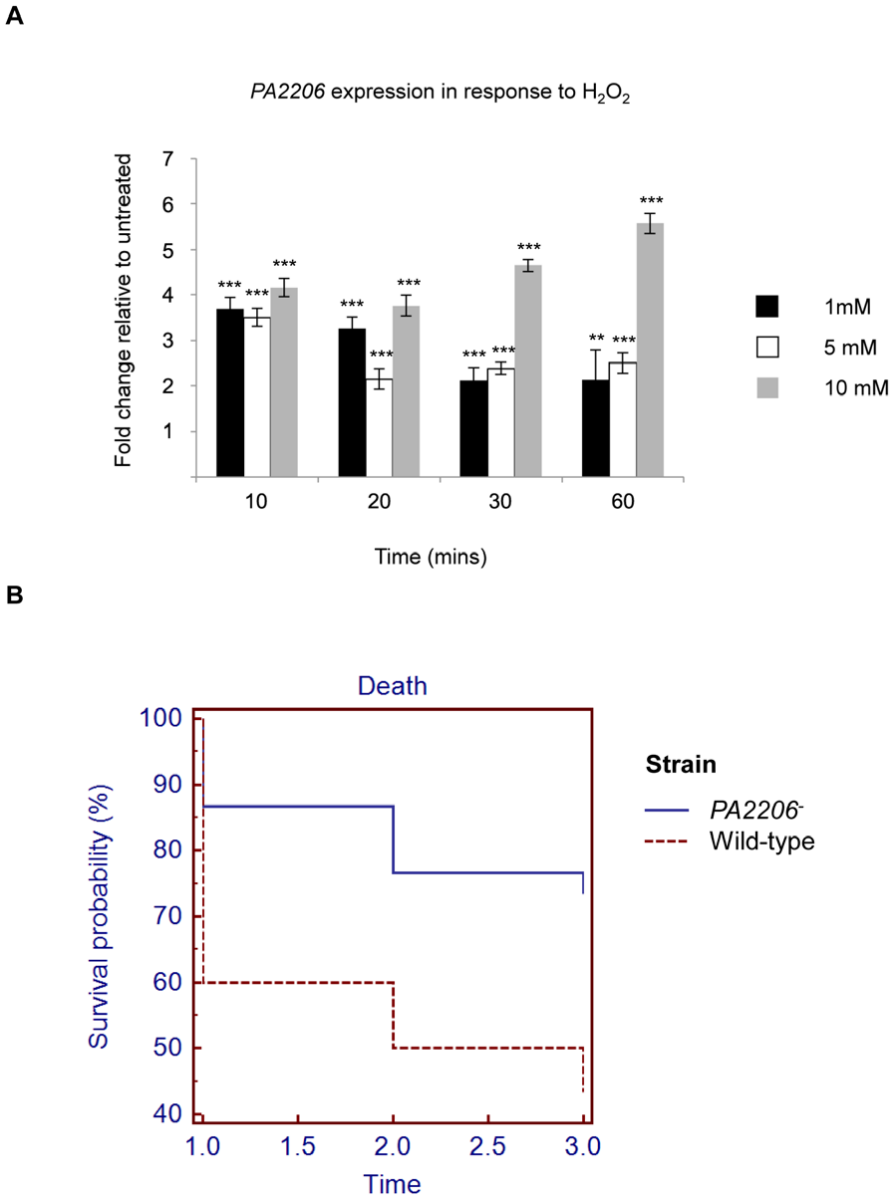
PA2206 is induced in response to H_2_O_2_ and is required for lethality in a zebrafish embryo model of infection. (**A**) Bacterial cultures were grown to exponential phase, and aliquots were then either untreated or exposed to 1 mM, 5 mM and 10 mM concentrations of H_2_O_2_. *PA2206* gene expression was measured as fold change relative to the *proC* housekeeping gene and subsequently calculated as fold change relative to the untreated sample, following 10, 20, 30, and 60 minute periods of exposure to H_2_O_2_. Mean values are represented ± standard error (** p-value≤0.005, *** p-value≤0.001 calculated using one-way ANOVA). (**B**) Kaplan-Meier survival curve. Twenty-six hours Zebrafish embryos were injected with ∼300 colony forming units of either mPAO1 wild-type or *PA2206^−^* mutant into their blood island. Embryos were monitored for survival daily. Mean values of three biological experiments (each 10 embryos) are shown. Statistical analysis showed that at 2 and 3 days post infection, mPAO1 wild-type killed significantly more embryos compared to the *PA2206^−^* mutant (p-value = 0.0159 using the log-rank test).

To confirm the induction of *PA2206* in response to oxidative stress, the kinetics of *PA2206* expression in the presence of H_2_O_2_ was investigated. Quantitative Real Time RT-PCR analysis demonstrated that expression of *PA2206* was induced in response to sublethal 1, 5, and 10 mM concentrations of H_2_O_2_, following exposure for 10, 20, 30, and 60 minute time periods (Fig. 1A). Expression of *PA2206* remained elevated (2–4 fold) in a dose dependent manner for up to an hour after exposure to H_2_O_2_ with the greatest induction observed in response to 10 mM H_2_O_2_ (Fig. 1A). In order to assess whether this induction was specific for H_2_O_2_, expression was investigated in the presence of other oxidative stress inducing compounds. Exposure to the superoxide generator menadione (2-methyl-1,4-naphthoquinone) resulted in 1.7-fold (±0.15) induction of *PA2206* gene expression (Fig. S2), while the organic hydroperoxides cumene hydroperoxide and tert-butyl hydroperoxide did not influence expression of PA2206 (data not shown) suggesting a degree of specificity to the activation of *PA2206* in response to oxidative stress.

To investigate the induction of *PA2206* at the transcriptional level, two possibilities were considered; *PA2206* may be (1) autoregulatory in the presence of a signal generated during oxidative stress, or (2) under the control of another transcriptional regulator. Although several putative LysR boxes were identified upstream of the *PA2206* gene, promoter fusion analysis revealed that mutation or overexpression of *PA2206* had no significant influence on its own promoter activity, with comparable Miller Units detected in wild-type, *PA2206^−^* and complemented *PA2206^C^* strains (Fig. S3). This suggested that PA2206 was not autoregulatory, again in contrast to the classical mechanism of LysR regulation. To investigate whether induction of *PA2206* was the result of regulation by OxyR (the master oxidative stress regulator), expression of *PA2206* was investigated using qRT-PCR in wild-type and in the *oxyR* mutant grown in M9 media supplemented with 2% (w/v) glucose. *PA2206* was induced to comparable levels in the wild-type and *oxyR* mutant following exposure to 5 mM or 10 mM H_2_O_2_ (Haynes & O'Gara, unpublished data) suggesting that PA2206 expression is induced independently of OxyR during the oxidative stress response.

### PA2206 is required for an efficient oxidative stress response and pathogenesis in *P. aeruginosa*

In order to determine whether the induction of *PA2206* in response to H_2_O_2_ and menadione correlated with a stress-tolerant phenotype, growth and viability of the wild-type and *PA2206* ^−^ mutant strains in the presence of exogenous ROS was investigated. Although growth in the untreated samples was comparable for mutant and wild-type strains, growth was significantly reduced in the *PA2206^−^* mutant compared to wild-type upon addition of H_2_O_2_ (20 mM) or menadione (40 mM) to exponentially growing cells (Fig. S4). In addition, time-kill survival assays provided further evidence that *PA2206^−^* mutant was hypersensitive to H_2_O_2_ (Table 2). Viable counts for mutant and wild-type strains determined before (time 0) and one hour after (time 1) exposure to H_2_O_2_ (a 1 hr sampling time was chosen to assess the steady state response rather than the early and acute response) revealed a significant reduction in survival in the *PA2206^−^* mutant compared to the wild-type strain (Table 2). In contrast, wild-type cells were able to survive exposure to this physiologically high concentration (100 mM). Introduction of *PA2206 in trans* (*PA2206^C^*) restored H_2_O_2_ tolerance of the mutant to 80% of the wild-type strain. Taken together, these data demonstrated that the *PA2206^−^* mutant was significantly more susceptible than the wild-type to exogenous ROS.

**Table 2:** Susceptibility of wild-type, *PA2206^−^* and *PA2206* ^C^ strains to oxidative stress.

Strain	Viable counts (cfu/ml) (×10^6^)			% Survival
	Time 0 (hours)		Time 1 (hours)	
Wild-type	58±2.7		70±3.0	100
*PA2206^−^*	58±9.0		34±4.2	47
*PA2206^C^*	54±3.5		51±4.2	79

Mean values are represented ± standard deviation.

To assess the *in vivo* relevance of the oxidative stress sensitive phenotype of the *PA2206^−^* mutant, lethality in the zebrafish embryo model of infection was investigated. Several reports suggest that some transcriptional regulators that influence the oxidative stress response can have a significant impact on pathogenesis or lethality in animal models while others exhibit no influence [38], [39], [40], [41]. Consistent with the stress-susceptible phenotype of *PA2206^−^*, the mutant exhibited significantly less killing than the wild-type in the zebrafish embryo model. Killing was reduced to 72% at 2 days post infection (d.p.i.) and 60% (3 d.p.i.) when compared to wild-type (Fig. 1B). Indeed, embryo survival remained constant at 90% in the presence of the mutant strain throughout these experiments. Interestingly, cytotoxicity, invasion and adhesion in epithelial cells was comparable between wild-type and *PA2206^−^* (data not shown). Therefore, to better understand the molecular mechanism underpinning the oxidative stress and zebrafish lethality phenotypes of the *PA2206^−^* mutant, we undertook global transcriptomic profiling.

### Transcriptomic analysis reveals a global profile for PA2206

**Figure 2:**
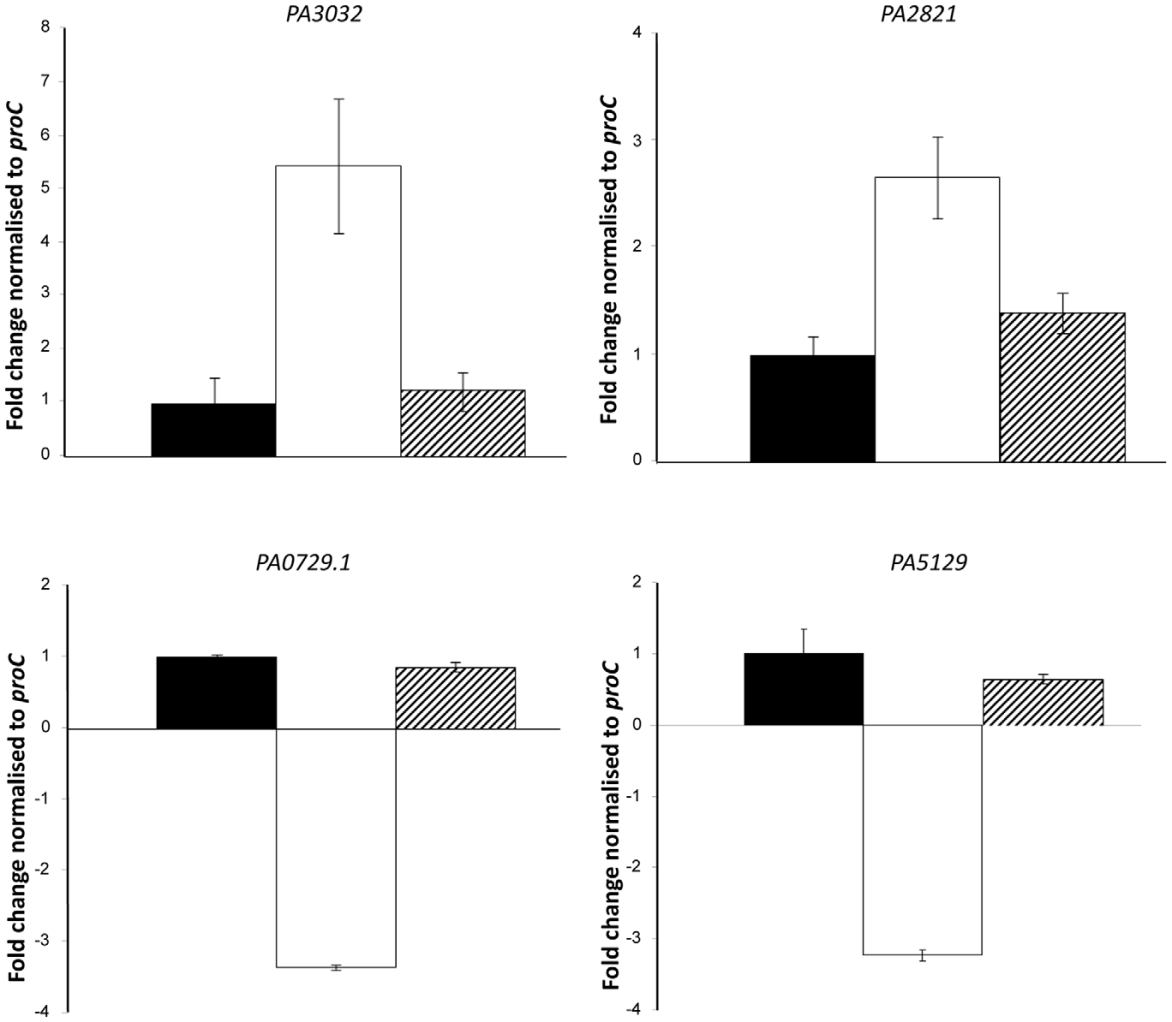
Quantitative Real Time PCR analysis confirms the influence of PA2206 on gene expression linked to the oxidative stress response. Bacterial cultures were grown to exponential phase, and aliquots were then either untreated or exposed to a 1 mM concentration of H_2_O_2_ for 10 mins. Expression of *PA3032*, *PA2821*, *PA0729.1* and *PA5129* was measured as fold change relative to the *proC* housekeeping gene. Mean values are represented ± standard error. Consistent with the array data, expression of *PA3032* and *PA2821* was increased in the *PA2206^−^* mutant (white bar) compared to *PA2206^C^* (striped bar) and the wild-type (black bar) strain. Similarly, expression of *PA0729.1* and *PA5129* was approximately 3-fold less in the *PA2206^−^* mutant strain (p-value of <0.05 by student's ttest).

In order to uncover the extent of the PA2206 transcriptional regulon during oxidative stress, gene expression profiles were compared between isogenic *PA2206* ^C^ and *PA2206* ^−^ in response to a sub-lethal 1 mM concentration of H_2_O_2_. This sub lethal concentration was chosen as it most accurately reflects the physiological levels found in the CF lung and other sites where the PMN inflammatory response occurs (33). Approximately 1% of genes (58 of the 5,500 *P. aeruginosa* PAO1 probe sets on the microarray) demonstrated significantly (>2-fold) altered expression (Table 3). Of these, 34 genes were upregulated and 24 genes were downregulated in *PA2206* ^C^ relative to *PA2206* ^−^, and the changes in gene expression ranged from +7.7 to −3.4 fold (Table 3). The dataset was validated by qRT-PCR, including the wild-type strain for comparison. Expression of the *grx* glutaredoxin (*PA5129*) and a glycine tRNA (PA0729.1) was decreased in the *PA2206* mutant, while a glutathione S-transferase (*PA2821*) and the *snr* cytochrome (*PA3032*) were found to be increased (Fig. 2). These results were in accordance with the microarray analysis.

**Table 3:** Microarray analysis of the transcriptional influence of *PA2206* in the presence of hydrogen peroxide.

ORF ID	Gene name	Description	Fold change
*Genes positively regulated by PA2206*			
PA0321		probable acetylpolyamine aminohydrolase	3.8
PA0323		spermidine/putrescine-binding periplasmic protein	2.0
PA0472	*fiuI*	probable sigma-70 factor, ECF subfamily	2.0
PA0609	*trpE*	anthranilate synthetase component I	3.0
PA0649	*trpG*	anthranilate synthase component II	2.4
PA0729.1		tRNA Glycine	2.8
PA1409	*aphA*	acetylpolyamine aminohydrolase	6.1
PA1410		periplasmic spermidine/putrescine-binding protein	4.3
PA1912	*femI*	ECF sigma factor, FemI	2.4
PA1961		probable transcriptional regulator	2.2
PA2033		hypothetical protein	2.2
PA2206		probable transcriptional regulator	6.4
PA2214		major facilitator superfamily (MFS) transporter	2.2
PA2215		hypothetical protein	3.8
PA2216		hypothetical protein	2.7
PA2426	*pvdS*	sigma factor	5.3
PA2594		hypothetical protein	3.3
PA2761		hypothetical protein	2.2
PA3229		hypothetical protein	3.0
PA3515		hypothetical protein	2.0
PA3516		probable lyase	2.5
PA3517		probable lyase	2.0
PA3530		hypothetical protein	2.6
PA4515		uncharacterized iron-regulated protein	3.0
PA4516		hypothetical protein	2.5
PA4659		probable transcriptional regulator	2.2
PA4660	*phr*	deoxyribodipyrimidine photolyase	2.2
PA4695	*ilvH*	acetolactate synthase isozyme III small subunit	2.1
PA4708	*phuT*	haeme-transport protein	2.0
PA4709		probable hemin degrading factor	2.5
PA4773		hypothetical protein	2.0
PA4881		hypothetical protein	7.7
PA5129	*grx*	glutaredoxin	2.2
PA5217		binding protein component of ABC iron transporter	3.1
*Genes negatively regulated by PA2206*			
PA0105	*coxB*	cytochrome c oxidase, subunit II	0.37
PA0106	*coxA*	cytochrome c oxidase, subunit I	0.37
PA0788		hypothetical protein	0.36
PA1216		hypothetical protein	0.35
PA1217		probable 2-isopropylmalate synthase	0.50
PA1317	*cyoA*	cytochrome o ubiquinol oxidase subunit II	0.47
PA2166		hypothetical protein	0.29
PA2747		hypothetical protein	0.42
PA2821		probable glutathione S-transferase	0.38
PA3032	*snr1*	cytochrome c Snr1	0.29
PA3361	*lecB*	fucose-binding lectin PA-IIL	0.51
PA3369		hypothetical protein	0.36
PA3370		hypothetical protein	0.32
PA3371		hypothetical protein	0.35
PA3416		pyruvate dehydrogenase E1 component, β chain	0.48
PA3417		pyruvate dehydrogenase E1 component, α subunit	0.50
PA3451		hypothetical protein	0.50
PA3788		hypothetical protein	0.40
PA4141		hypothetical protein	0.50
PA4738		conserved hypothetical protein	0.44
PA4739		conserved hypothetical protein	0.48
PA5085		probable transcriptional regulator	0.31
PA5481		hypothetical protein	0.39
PA5482		hypothetical protein	0.37

Genes that exhibited a 2-fold or greater alteration in expression in the *PA2206^C^* strain compared to the *PA2206^−^* mutant strain (p<0.05) are listed. Positive values indicate those genes which were upregulated in the *PA2206^C^* strain while values less than 1.0 indicate those genes which were downregulated.

**Figure 3:**
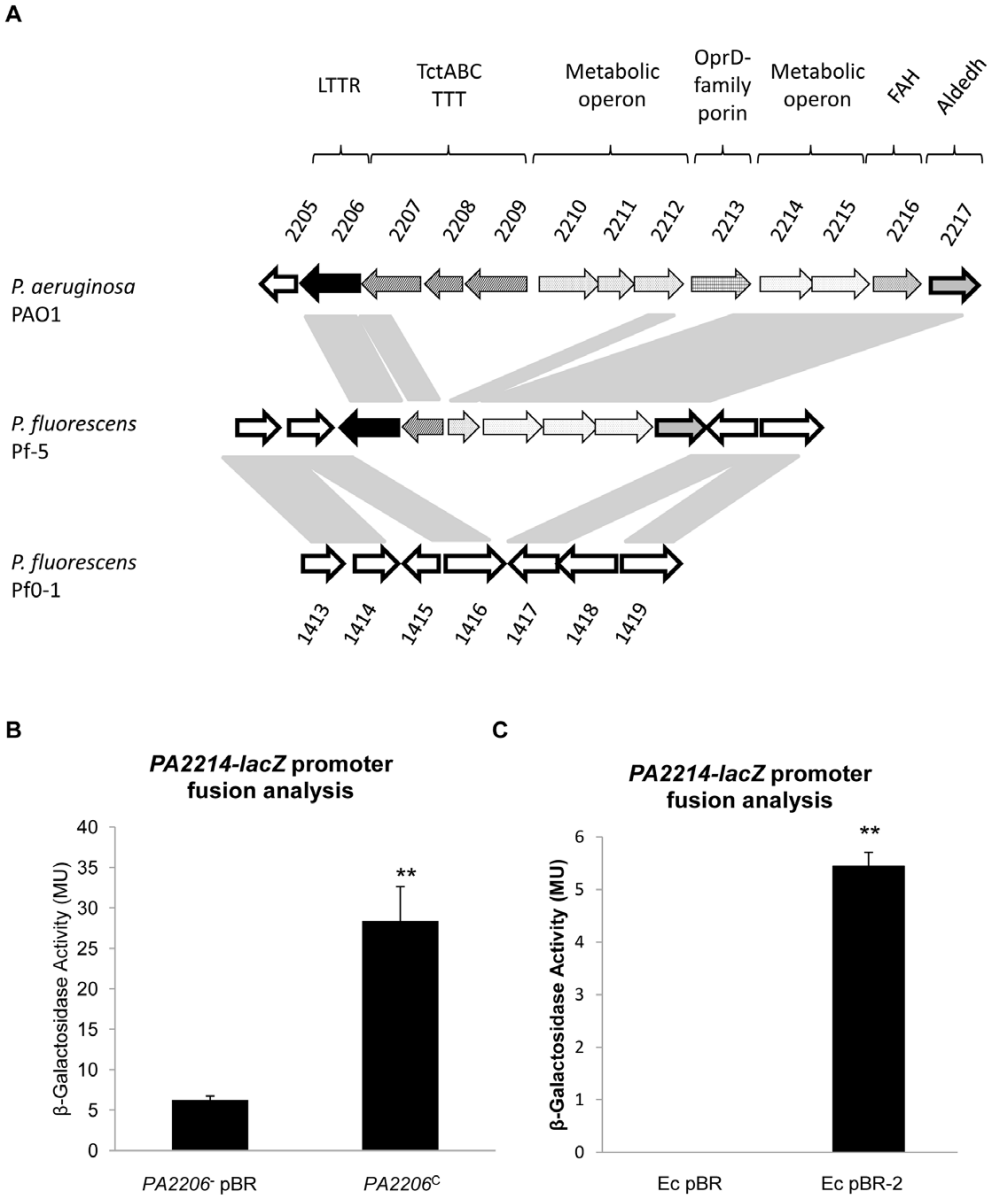
*PA2214-15* is under the direct transcriptional control of the PA2206 LysR regulator. (**A**) Comparative genomic analysis of the metabolic-centric *PA2206* region in *P. aeruginosa* PAO1, *P. fluorescens* Pf-5, and *P. fluorescens* Pf-O1 based on the Pseudomonas Genome Database. Single genes and operons are denoted by colour and pattern fill while homology is denoted by connecting shaded regions. *P. fluorescens* encodes a *PA2206* homologue, which is adjacent to truncated genes corresponding to fragments of *PA2207* and *PA2212*, both of which are downstream of a conserved *PA2214-2216* homologous operon. TTT denotes a tripartite tricarboxylate transport system, FAH denotes a putative fumarylacetoacetate hydrolase activity, while Aldedh denotes a putative aldehyde dehydrogenase activity. (**B**) Promoter-fusion analysis of the *PA2214* upstream region revealed significantly increased promoter activity in the *PA2206^C^* strain relative to *PA2206^−^*. The increase in promoter activity was consistently observed in three independent experiments consisting of three biological replicates (** p-value<0.01 by student's ttest). (**C**) *PA2214-lacZ* promoter fusion analysis performed in *E. coli* harbouring a *PA2206*-pBBR1MCS5 construct compared to vector control revealed direct regulation. Promoter activity was below baseline in the vector control (compared to empty pBBR1MCS5 plasmid), while an average of 6 Miller Units was consistently detected in the overexpressing strain (** p-value of <0.01 by student's ttest).

Consistent with promoter fusion and expression analysis (data not shown), the genes adjacent to *PA2206* were unaltered in the transcriptome profile. However, expression of the distantly encoded *PA2214-15* operon was increased 2–3 fold in the *PA2206* ^C^ strain as was expression of *PA2216* (Table 3). Comparative genomic analysis of publically available genome sequences revealed the *PA2206-2217* region to be exclusive to *P. aeruginosa*, while truncated segments were encoded in the *P. fluorescens* Pf-5 and *P. fulva* 12× genomes (Fig. S5). Intriguingly, homologues of the *PA2214-17* genes were identified adjacent to the *PA2206* homologue in *P. fluorescens* Pf-5 (Fig. 3A). Furthermore, hydrogen peroxide sensitivity assays revealed comparable zones of inhibition in *PA2206* and *PA2215* mutants (a *PA2214* mutant was not available from the mutant collection), both of which were significantly more sensitive than wild-type (Table S2). Therefore, to investigate whether these loci were indeed direct targets of the PA2206 LTTR, promoter fusion and DNA-binding analyses were undertaken.

### PA2206 positively controls *PA2214-15* expression

The genomic organisation and transcriptomic data suggested *PA2214-15* and/or *PA2216* promoters may be direct targets of PA2206 regulation. To investigate this hypothesis, transcriptional reporter fusions were introduced into the *PA2206^−^* and *PA2206^C^* strains. β-Galactosidase assays revealed a significant increase in *PA2214-15* promoter activity in the *PA2206^C^* (28.6 MU±8.0) strain compared to *PA2206* ^−^ (6.2 MU±0.8) (Fig. 3B). *PA2216* promoter activity was not significantly different in both strains; *PA2206^C^* (128 MU±17.0) and *PA2206^−^* (123 MU±8.7). This suggested that the *PA2214-15* promoter was under the transcriptional control of PA2206.

The *PA2214-lacZ* transcriptional reporter fusion was subsequently introduced into *E. coli* harbouring a pBBR1MCS plasmid constitutively expressing *PA2206* (EcpBR-2) with the aim of elucidating whether the *PA2214-15* promoter was a direct target of PA2206. As *E. coli* does not encode a *PA2206* homologue, activation of the *PA2214-15* promoter in this strain would most likely be the result of a direct interaction between the LysR protein and the promoter DNA. *PA2214-15* expression was significantly induced upon overexpression of *PA2206*, while promoter activity was not detected in the vector control suggesting that this promoter is under the direct transcriptional control of *PA2206* (Fig. 3C). To confirm this direct interaction, DNA-mobility shift assays were performed.

### PA2206 binds adjacent to the putative −35 region of the *PA2214-15* promoter

**Figure 4:**
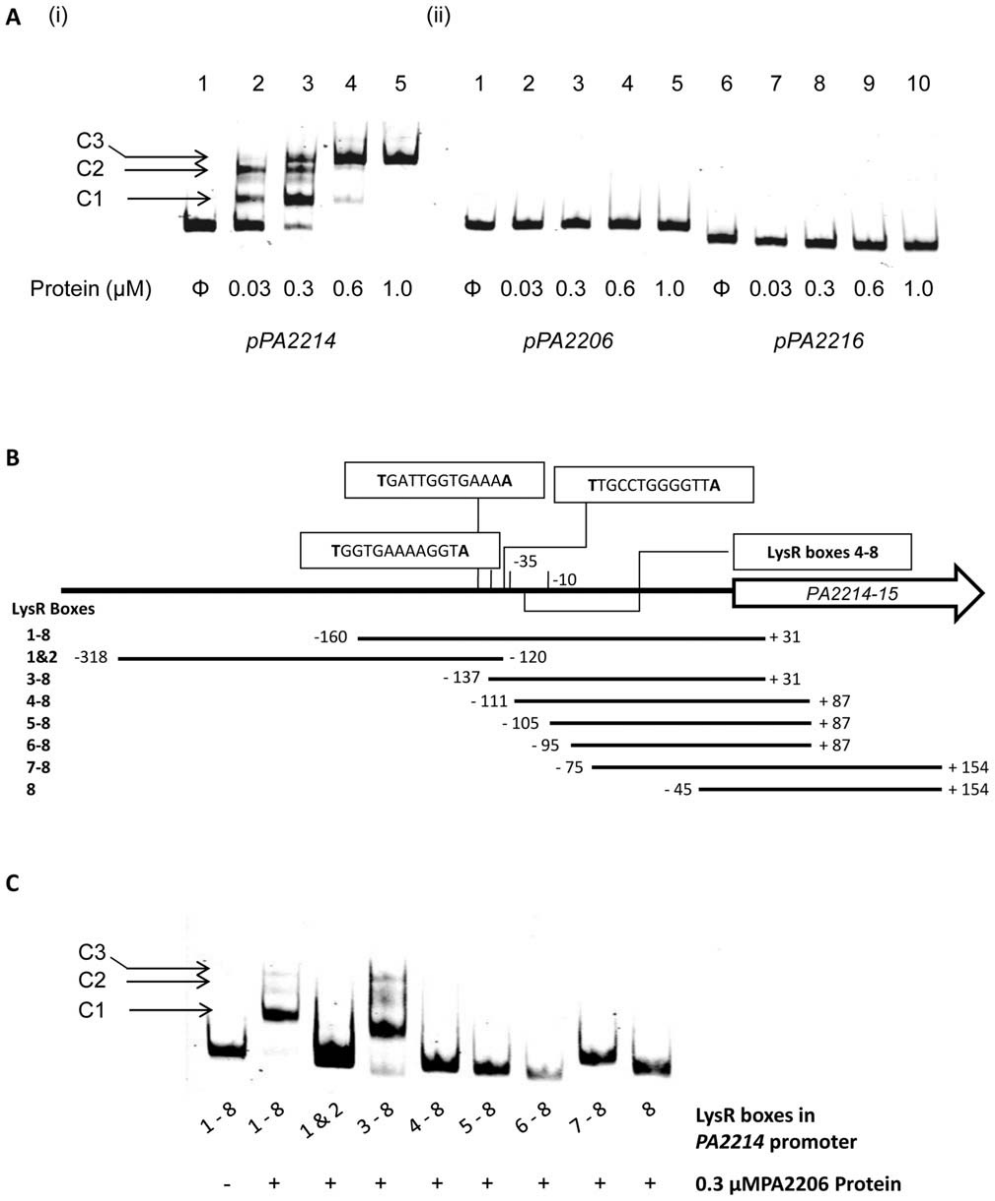
PA2206 binds a LysR-box overlapping the predicted −35 box in the *PA2214* promoter. (**A**) EMSA analysis of the *PA2214-15*, *PA2206* and *PA2216* promoter fragments revealed *PA2214-15* to be a direct PA2206 target. The protein concentration is marked below each lane while DNA promoter fragments were used at 15 fmoles. Three complexes were observed upon protein interaction with the *PA2214-15* (1–3) promoter fragment at low nanomolar protein concentrations, with the C2/C3 complexes predominant at higher concentrations of PA2206 protein. The *PA2206* (4–6) and *PA2216* (7–9) promoter fragments did not form a complex with the PA2206 protein. (**B**) Schematic diagram of the location and arrangement of the LysR boxes identified upstream of the predicted transcriptional start site of *PA2214*. The primer positions for each of the truncated promoter fragments are outlined below and aligned with the LysR boxes contained within each amplicon. (**C**) Mobility shift analysis of the PA2206 protein interaction with the *PA2214* promoter region. Protein was added to each reaction at 300 nM and DNA promoter fragments were used at 15 fmoles. The strong shift in lane 4 confirms that PA2206 binds to LysR box 3 overlapping the p*PA2214* predicted −35 region and that this region is sufficient for the interaction to occur. PA2206 protein did not cause a shift for any of the other truncated promoter fragments.

In order to provide direct evidence of the PA2206 binding interaction with the *PA2214* promoter, a HisTag PA2206 construct was generated and introduced into *E. coli* BL21 DE cells for protein expression. After IPTG induction and purification through a nickel affinity column, protein purity and concentration were determined by SDS PAGE analysis and Bradford assay, respectively. Subsequent EMSA analysis revealed an interaction at the low nanomolar range of PA2206 protein with the −160 to +31 (relative to start of translation) region of the *PA2214-15* promoter, amplified using the PA2214F and PA2214R primers (Fig. 4A and Table S1). Three complexes between the PA2206 protein and the *PA2214* promoter were observed, formed in a concentration dependent manner (Fig. 4). A similar stoichiometry has previously been reported for other LTTRs, notably CysB from *Salmonella enterica* [42]. No such interaction was observed at the −246 to −14 region of the *PA2206* promoter (consistent with the lack of autoregulation observed at the transcriptional level) or the −278 to −106 region of the *PA2216* promoter (Fig. 4A). Taken together with the promoter fusion analysis (Fig. 3), this would appear to confirm *PA2214-15* as a direct target of PA2206 regulation.

Truncated *PA2214* promoter fragments were generated with the aim of identifying the specific LysR motif with which the PA2206 protein interacts. The region of the *PA2214* promoter to which PA2206 was shown to bind contains five putative LysR boxes upstream of the predicted (Bprom) transcriptional start site (Fig. 4B). Box 3 is situated adjacent to the predicted −35 region, while boxes 1 and 2 overlap each other directly upstream. Boxes 4 and 5 are situated between the predicted −35 and −10 regions, while a further three LysR boxes (boxes 6–8) were situated between the putative transcriptional and translational start sides. EMSA analysis subsequently revealed that PA2206 formed a complex with the promoter fragment containing boxes 3–8 but did not bind to the fragment containing boxes 1 & 2 (Fig. 4C). Neither did PA2206 bind to the promoter fragments located downstream of box 3 suggesting that this motif alone is sufficient for direct contact with PA2206 (Fig. 4C). Although F2-TrR (boxes 1&2) overlaps the 5′ ATTGCC region of LysR box 3, and TrAF-TrR (boxes 4–8) is directly adjacent to the 3′ region, no interaction with PA2206 was observed with these fragments, further evidence that the complete 5′-ATTGCCTGGGGTTAT-3′ LysR box is required for interaction with PA2206. It is worth noting that this sequence is highly similar to the ATAACC-N4-GGTTAT motif reported for the PcaQ LysR transcriptional regulator from *Sinorhizobium meliloti* [43], [44], consistent with the *PA2206* LysR domain annotation. The emerging global dimension to LysR regulation suggested that there may be additional targets of PA2206 regulation in *P. aeruginosa*. Therefore, BioPerl analysis was undertaken to identify the extent of the PA2206 regulon.

### PA2206 has additional promoter targets suggesting a global regulatory role

**Figure 5:**
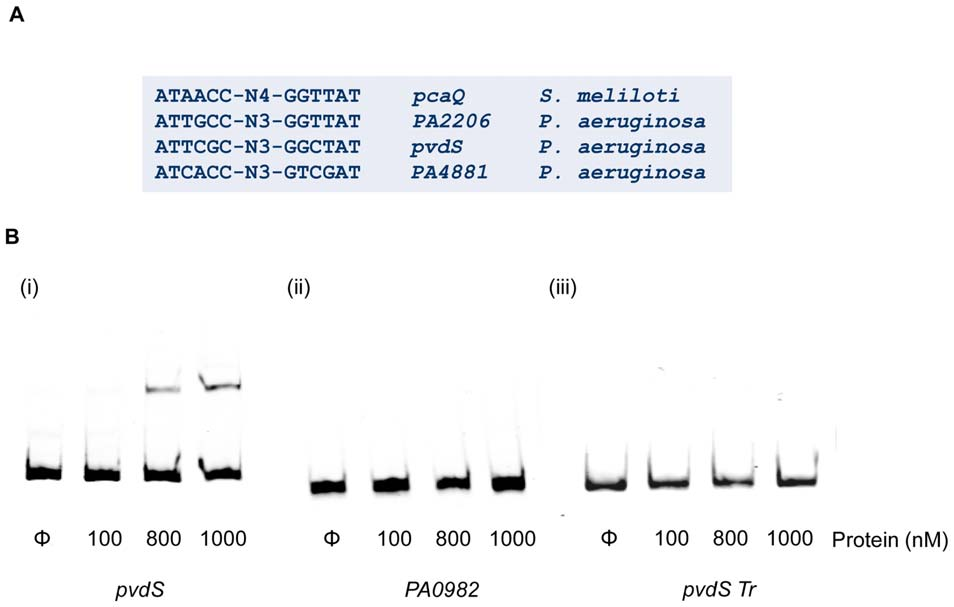
PA2206 has a global regulatory role in *P. aeruginosa*. (**A**) BioPerl analysis revealed the presence of the putative PA2206 consensus sequence in the promoters of the *pvdS* and *PA4881* genes. The arrangement and sequence similarity of the consensus sequence was similar to that of the *pcaQ* LTTR from *S. meliloti*. (**B**) (i) EMSA analysis of the *pvdS* promoter region with the PA2206 protein revealed an interaction at nanomolar concentrations. Concentrations as low as 800 nM were able to shift the *pvdS* promoter. (ii) The absence of binding to the *PA0982* promoter probe indicates the requirement for specificity in the predicted consensus sequence. (iii) No interaction was observed between PA2206 and a truncated *pvdS* promoter fragment located downstream of the putative LysR box suggesting that this sequence alone is sufficient for the protein-DNA interaction to occur.

To investigate whether PA2206 has a global regulatory role, a BioPerl script was generated to screen the PAO1 genome sequence for LysR consensus sequences with similarity to the 5′-TTGCCTGGGGTTA-3′ sequence of the *PA2214-15* promoter. The absence of PA2206 homologues from species other than *P. aeruginosa*, with few exceptions, hindered the generation of a stringent consensus sequence. Furthermore, site-directed mutagenesis will be required to identify the nucleotides that are critical for PA2206 binding. However, LysR boxes with close sequence identity to the PA2206 LysR box were identified in 18 unique promoters (Fig. S6). Cross-referencing of this list with the transcriptomic data revealed several potential targets including the *pvdS* (*PA2426*, 5.26-fold upregulated) and *PA4881* (7.66-fold upregulated) promoters. The *pvdS* promoter was chosen for further investigation on the basis of the relationship between iron metabolism and oxidative stress, and EMSA analysis revealed a specific interaction with the PA2206 protein in the nanomolar range (Fig. 5B). In contrast to the interaction of PA2206 with the *PA2214* promoter (C1–C3 complexes, Fig. 4), the *pvdS* interaction occurred as a single complex migrating similar to the C2/C3 complex, with this differential stoichiometry possibly reflecting distinct affinities or oligomeric binding arrangements at each promoter [45]. This same pattern of interaction has previously been reported for the binding interaction of other LTTRs with target promoters, including that of the CatR LTTR to the *catBC* and *clcABD* promoters [12]. Binding analysis using a truncated *pvdS* promoter fragment downstream of the putative PA2206 box revealed no interaction suggesting that PA2206 binds to the identified consensus sequence in the *pvdS* promoter (Fig. 5B). In addition, PA2206 was also found to bind to the *PA4881* promoter, consistent with its upregulation in the transcriptomic profile, forming C1 and C2/C3 complexes in a concentration dependent manner (Fig. S6). The specificity of these interactions was highlighted by the lack of any mobility shift with the PA0982 (Fig. 5B) and PA1874 (Fig. S6) promoters. Regulation of *pvdS* and *PA4881* suggests a global role for PA2206 in *P. aeruginosa*, although further analysis incorporating Co-immunoprecipitation (Co-IP) analysis will be required to define the full repertoire of PA2206 targets in the cell. Surprisingly, neither *pvdS* nor *PA4881* exhibited increased susceptibility to hydrogen peroxide using simple disk diffusion assays (data not shown). Therefore, the mechanism through which PA2206 contributes to the oxidative stress response in *P. aeruginosa* may involve a more complex interplay between downstream targets of PA2206.

## Discussion

Microbial pathogens are continuously faced with the threat of oxidative stress elicited by the host immune response, and also from the by-products of aerobic metabolism. Their ability to sense and rapidly respond to oxidative stress arising from elevated levels of ROS is central to their pathogenesis and survival. For example, the presence of pathogens such as *P. aeruginosa* in the human lower respiratory tract leads to the induction of host-derived mediators such as pro-inflammatory cytokines and chemokines which activate phagocytic cells including alveolar macrophages and neutrophils [46], [47]. The phagocytes target bacterial populations with the production of oxidative stress inducing reactive oxygen species (ROS), proteolytic enzymes and a range of toxic metabolites [48]. As a result *P. aeruginosa* has evolved a complex repertoire of strategies to overcome the deleterious effects of these and other toxic intermediates.

The response to oxidative stress is complex and highly coordinated, involving enzymatic and non-enzymatic activities and requiring multiple layers of regulation. In this study, PA2206 has been identified as an important component of transcriptional regulation required for the oxidative stress response and zebrafish lethality in *P. aeruginosa*. The dual involvement of transcriptional regulators in the oxidative stress response and pathogenesis has previously been reported. For example, OxyR is required for virulence of *P. aeruginosa* in insect and mouse models of infection [39] and also for secretion of cytotoxic factors [49]. In addition to this, Lan and colleagues demonstrated that another oxidative stress response regulator, OspR, influences levels of dissemination of *P. aeruginosa* in a murine model of acute pneumonia, with inactivation of OspR resulting in increased bacterial virulence [38]. In contrast, a study carried out in *S. enterica* serovar Typhimurium demonstrated that neither OxyR nor KatG (catalase) influence survival in the presence of neutrophils [40], while AhpC (peroxidise) is not required for virulence in a mouse model [41]. Therefore, microbes may have evolved distinct roles for these transcriptional regulators, consistent with their ecological and niche-specific requirements. It remains to be determined whether the altered pathogenesis observed upon mutation of *PA2206* in the zebrafish embryo model of infection is a consequence of its reduced tolerance to oxidative stress or through independent alteration of its *in vivo* virulence profile.

The molecular mechanism underpinning the global regulatory role of PA2206 involves at least three direct targets; *PA2214-15*, *pvdS*, and *PA4881*, in addition to the possibly indirect regulation of 56 other genes during oxidative stress. *PA4881* remains uncharacterised, although it has recently been shown to encode a tandem repeat protein also under the control of the redox responsive LysR protein MexT [50], [51]. Annotated as hypothetical, the *PA2206-17* genomic region encodes a large number of genes encoding proteins with an apparent metabolic function. Furthermore, the positive regulation by PA2206 of several operons involved in polyamine metabolism may be significant in light of recent reports on the protective role of polyamines against oxidative stress in *E. coli* [52]. Re-routing metabolic flux has recently been proposed as a key mechanism through which OxyR mediates oxidative stress tolerance in *P. aeruginosa* [15]. Indeed, the flux of cellular metabolic pathways is emerging as a key adaptive mechanism through which both microbes and higher organisms adapt to the threat of ROS, although the molecular mechanisms governing these relationships remain to be defined [53], [54], [55], [56], [57]. It is worth noting the magnitude of the reduction in cell viability observed for the *PA2206^−^* mutant following exposure to H_2_O_2_ (Table 2) in comparison with the exquisite sensitivity following mutation of OxyR, which controls the battery of enzymes required to mount an acute response to oxidative challenge [14]. Mutation of *oxyR* in *P. aeruginosa* and other pathogens has been shown to result in an inability to grow on LB agar plates, possibly due to the autoproduction of concentrations as low as 1.2 µM H_2_O_2_ per minute [39], [58]. In addition, micromolar concentrations of H_2_O_2_ were sufficient to arrest the growth of *oxyR* mutants in several microbial species reinforcing its position as the master regulator of the oxidative stress response [59], [60]. Therefore, PA2206 may represent an important OxyR-independent regulatory link between perception of an exogenous stress and adaptation through realignment of lower metabolic pathways to conserve energy and facilitate survival.

The positive regulation of iron related genes by PA2206, including PvdS and both the FiuIRA and FemIRA ECF systems, supports a role for iron metabolism in the oxidative stress response in *P. aeruginosa*. Indeed, Palma and colleagues have previously reported induction of several iron-starvation responsive genes upon treatment with H_2_O_2_ suggesting either that cells experience iron starvation in response to oxidative stress, or that transient loss of Fur repressor function may occur [32], [33]. Furthermore, Wei and colleagues have also recently reported an OxyR-dependent link with iron homeostasis through positive regulation of *pvdS* during oxidative stress [15]. However, elucidating the full complexity of the role of iron uptake systems during oxidative stress will require further investigation. Notwithstanding this, the binding of PA2206 to the *pvdS* promoter in the nanomolar range, and the increased *pvdS* expression in the *PA2206^C^* strain, support a direct role for PA2206 in regulating iron homeostasis during oxidative stress. The concentration of PA2206 protein required to cause a mobility shift of the *pvdS* promoter was comparable to the recently reported regulation of the *pvdS* promoter by the LysR regulator of sulphur metabolism, CysB [61]. More recently, OxyR has also been shown to bind to the *pvdS* promoter [15] and regulation of the *pvdS* promoter by these three distinct LTTRs highlights the coordination of regulatory inputs that underpins microbial adaptation. However, although cross-talk among LTTRs may facilitate fine-tuning of the microbial stress response, it is important to distinguish the regulatory influences of OxyR and PA2206 during oxidative stress. While 28 genes that were either induced or repressed under conditions of oxidative stress [33] were differentially expressed in the *PA2206^C^* strain compared to an isogenic *PA2206* ^−^ mutant, only 6 of these loci were bound by OxyR in the recent study performed by Wei and co-workers [15]. Furthermore, in a separate study, proteomic analysis of the *oxyR* mutant under iron limitation [62] revealed no overlap with the *PA2206* transcriptomic dataset. Therefore, while transcriptomic analysis of the OxyR regulon will be required to fully appreciate the distinct patterns of regulation by OxyR and PA2206, our data would suggest that significant overlap would not be expected.

Complex adaptive responses such as oxidative stress and host pathogenesis require the input of several classes of regulatory protein, transcriptional, post-transcriptional and post-translational. The COG prediction of post-transcriptional regulation or post-translational modification for genes altered in the transcriptome, including glutathione S-transferase (*PA2821*), suggested that the oxidative stress phenotype associated with loss of PA2206 function may be multifactorial. Indeed, preliminary proteomic analysis has revealed reduced production of two probable peroxidases in the *PA2206^−^* mutant and time-kill survival assays revealed that both are required for survival in the presence of 100 mM H_2_O_2_ (Haynes & O'Gara unpublished data). As expression of the genes encoding these proteins was unaltered at the transcriptional level, the influence of PA2206 on these targets is likely to be indirect. Characterizing the molecular pathways that link the transcriptional and post-transcriptional systems identified in this study will provide the focus of future investigations.

In summary, PA2206 contributes to an effective oxidative stress response and pathogenicity in *P. aeruginosa*. The role of PA2206 during oxidative stress appears to be independent of the OxyR pathway, although cross-talk with other transcriptional regulators must be considered. Elucidating the molecular pathways underpinning this crucial aspect of microbial pathogenesis will provide a platform for greater understanding of the process of infection in *P. aeruginosa* and other serious pathogens.

## Supporting Information

Figure S1 **HisTag protein purification of PA2206.** Purified protein was loaded on an 10% SDS PAGE gel and visualised following Coomassie Blue staining. Imidazole concentrations used to elute each fraction are detailed below the gel.
(PPT)

Figure S2 ***PA2206*** **expression is induced in response to 1 mM menadione.** Bacterial cultures grown to exponential phase were exposed to a 1 mM concentration of menadione and expression of *PA2206* was found to be significantly increased. Mean values are represented +/− standard error.
(PPT)

Figure S3 ***PA2206*** **does not autoregulate its own promoter activity.** *PA2206-lacZ* promoter fusion analysis revealed no difference in β-galactosidase activity in wild-type, *PA2206* ^−^ or *PA2206^C^* strains. Data presented contains three biological replicates and is representative of three independent experiments (p-value≤0.01 by student's ttest).
(PPT)

Figure S4 **Growth profiling of wild-type and PA2206^−^ strains in the presence of ROS.** Addition of 20 mM H_2_O_2_ to exponentially growing cells led to significant reduction in growth rate in the *PA2206^−^* strain while the wild-type was unaffected. Similarly, addition of 40 mM menadione also resulted in reduced growth rate in the mutant strain. Significance of the growth impairment was confirmed by statistical analysis (Students ttest * p-value≤0.05, ** p-value≤0.005, *** p-value≤0.001).
(PPT)

Figure S5 **Comparative genomic analysis of the metabolic-centric** ***PA2206*** **region.** Analysis of all available *P. aeruginosa* genome sequences in which *PA2206* homologues were identified, including *P. fluorescens* Pf-5 and *P. fulva* 12×, based on the Pseudomonas Genome Database. Single genes and operons are denoted by colour and pattern-fill. Gene content and organisation is highly conserved in *P. aeruginosa* strains. *P. fluorescens* encodes a *PA2206* homologue, which is adjacent to truncated genes corresponding to fragments of *PA2207* and *PA2212*, both of which are downstream of a conserved *PA2214-2216* homologous operon. *P. fulva* encodes a TRAP system inserted between homologues of the *PA2213* and *PA2216* genes.
(PPT)

Figure S6 (**A**) Genome-wide BioPerl analysis of the 5′-TTGCCTGGGGTTA-3′ sequence revealed 18 promoters that contained putative LysR boxes with similarity (p<1e^−04^) to the PA2206 motif. MEME analysis of these LysR boxes led to the construction of a PA2206 consensus motif. Genes whose expression was altered in the PA2206^C^ strain are highlighted in bold and the fold changes are indicated in parentheses. (**B**) EMSA analysis reveals a binding interaction between PA2206 and the *PA4881* promoter, forming C1 and C2/C3 complexes in a concentration dependent manner. No interaction was observed at the PA1874 promoter, consistent with its lack of induction in the transcriptomic profile.
(PPT)

Table S1 **Primers used in this study.**
(DOC)

Table S2 **Disk diffusion analysis of the oxidative stress sensitivity of wild-type** ***P. aeruginosa*** **and** ***PA2206^−^*** **and** ***PA2215^−^*** **mutants.** Analysis performed using 10 µl of a 30% (w/w) solution of H_2_O_2_ (8.8 M).
(DOC)
