# Supplementary figures and images for: A Non-Classical LysR-Type Transcriptional Regulator PA2206 Is Required for an Effective Oxidative Stress Response in Pseudomonas aeruginosa
Source: PLoS One. 2013 Jan 28;8(1):e54479. doi: 10.1371/journal.pone.0054479 (PMC3557286; doi:10.1371/journal.pone.0054479)

## Slide 1
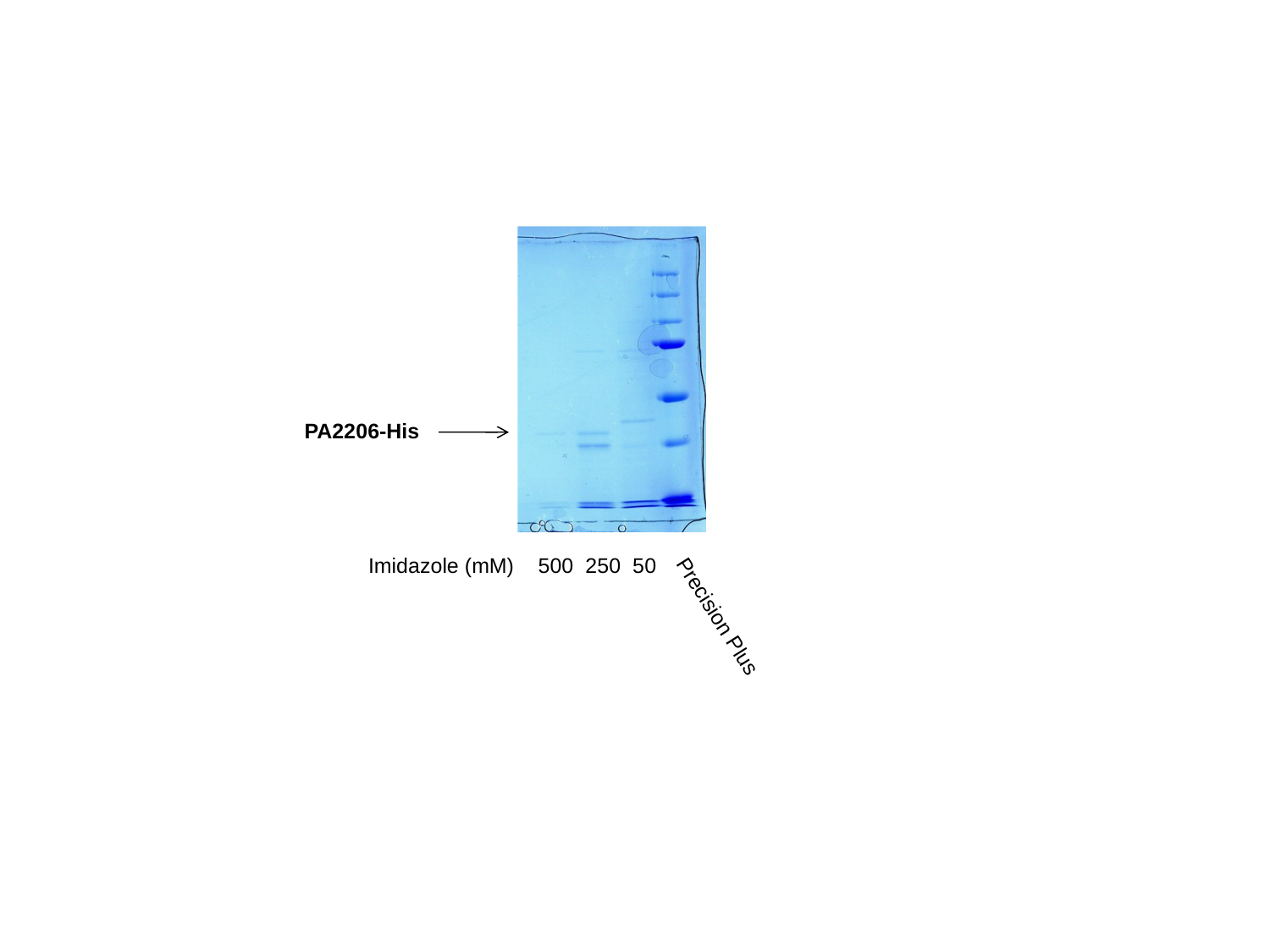

PA2206-His
Imidazole (mM)
500 250 50
Precision Plus

Supplement: Figure S1 — HisTag protein purification of PA2206. Purified protein was loaded on an 10% SDS PAGE gel and visualised following Coomassie Blue staining. Imidazole concentrations used to elute each fraction are detailed below the gel. (PPT) [file pone.0054479.s001.ppt]

## Slide 1
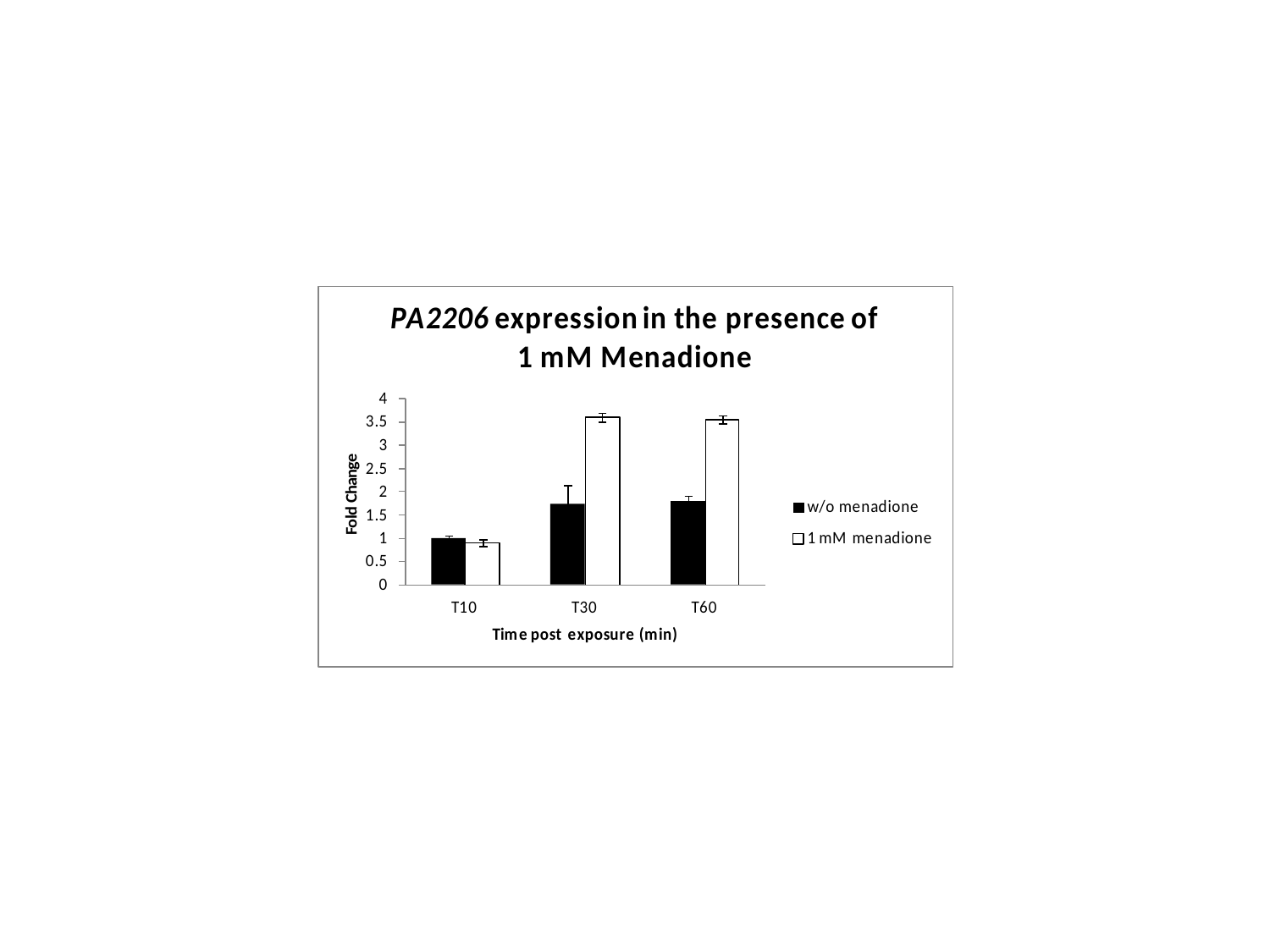

Supplement: Figure S2 — PA2206 expression is induced in response to 1 mM menadione. Bacterial cultures grown to exponential phase were exposed to a 1 mM concentration of menadione and expression of PA2206 was found to be significantly increased. Mean values are represented +/− standard error. (PPT) [file pone.0054479.s002.ppt]

## Slide 1
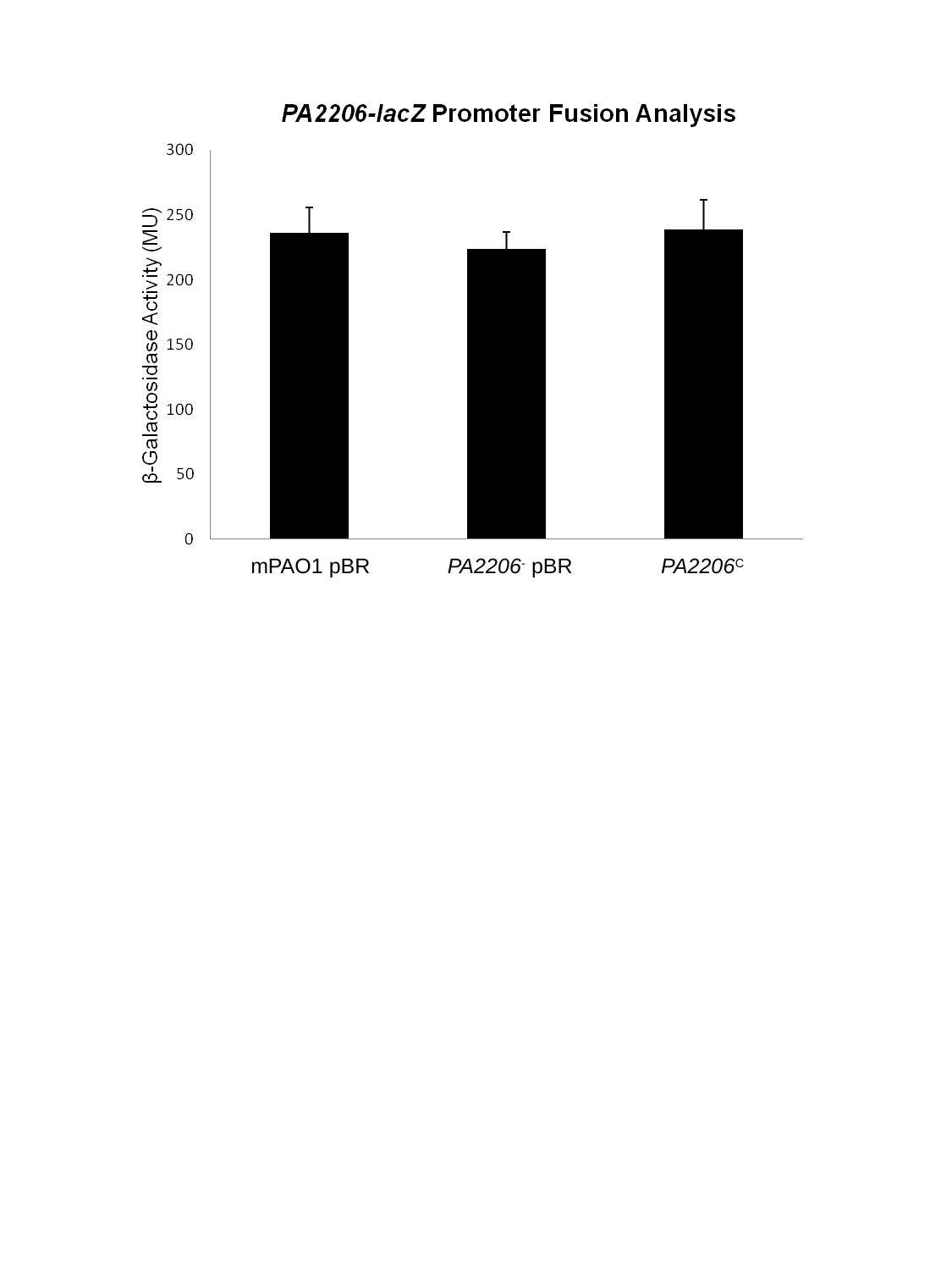

mPAO1 pBR PA2206- pBR	 PA2206C

Supplement: Figure S3 — PA2206 does not autoregulate its own promoter activity. PA2206-lacZ promoter fusion analysis revealed no difference in β-galactosidase activity in wild-type, PA2206 − or PA2206C strains. Data presented contains three biological replicates and is representative of three independent experiments (p-value≤0.01 by student's ttest). (PPT) [file pone.0054479.s003.ppt]
