# Supplementary material for: A Non-Classical LysR-Type Transcriptional Regulator PA2206 Is Required for an Effective Oxidative Stress Response in Pseudomonas aeruginosa
Source: PLoS One. 2013 Jan 28;8(1):e54479. doi: 10.1371/journal.pone.0054479 (PMC3557286; doi:10.1371/journal.pone.0054479)
Supplement: Figure S4 — Growth profiling of wild-type and PA2206− strains in the presence of ROS. Addition of 20 mM H2O2 to exponentially growing cells led to significant reduction in growth rate in the PA2206− strain while the wild-type was unaffected. Similarly, addition of 40 mM menadione also resulted in reduced growth rate in the mutant strain. Significance of the growth impairment was confirmed by statistical analysis (Students ttest * p-value≤0.05, ** p-value≤0.005, *** p-value≤0.001). (PPT) [file pone.0054479.s004.ppt]

## Slide 1
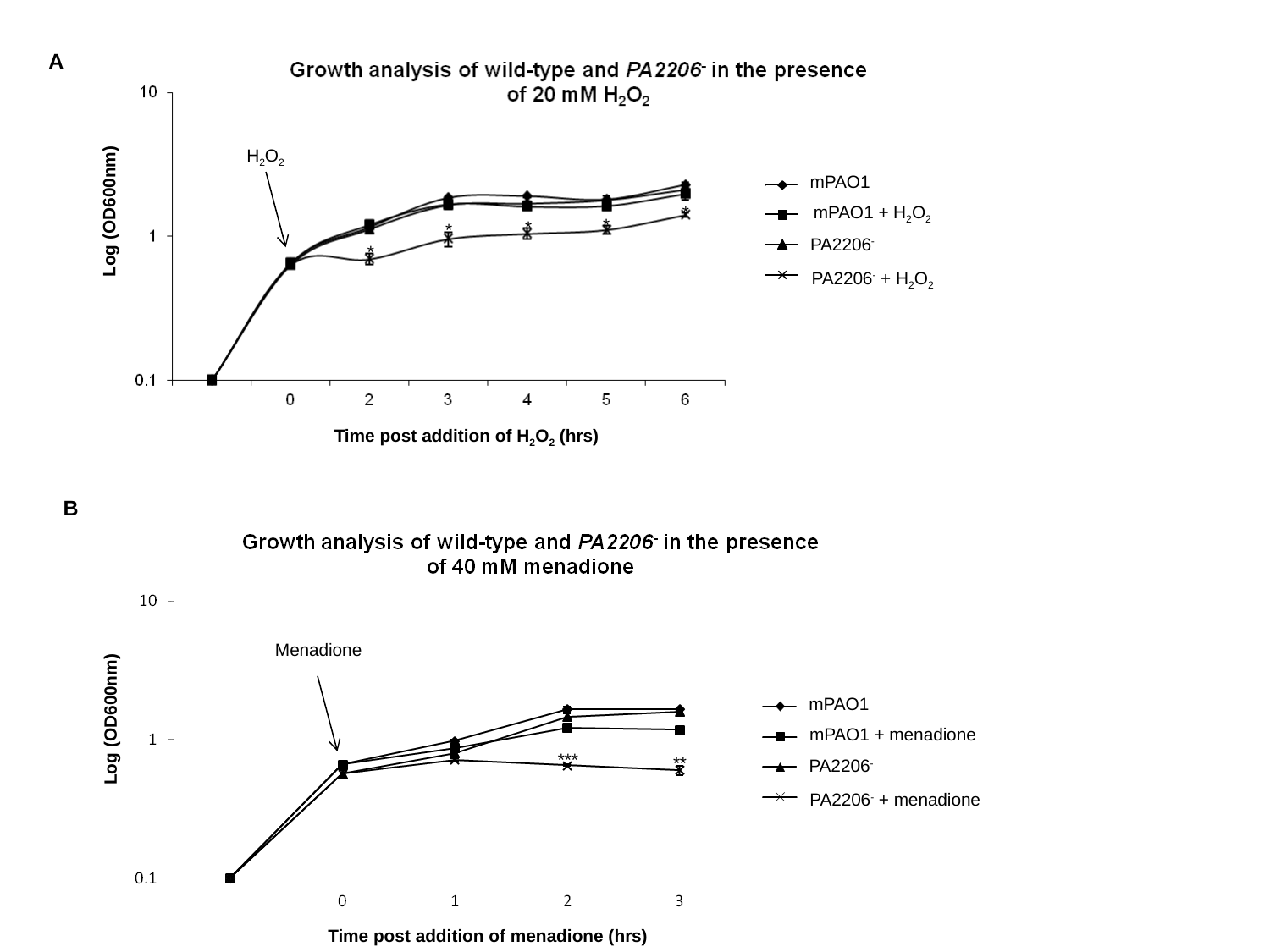

A
H2O2
Log (OD600nm)
Time post addition of H2O2 (hrs)
mPAO1
*
mPAO1 + H2O2
*
*
*
PA2206-
*
PA2206- + H2O2
B
mPAO1
mPAO1 + menadione
PA2206-
PA2206- + menadione
Menadione
Log (OD600nm)
Time post addition of menadione (hrs)
***
**
