# Supplementary material for: A Non-Classical LysR-Type Transcriptional Regulator PA2206 Is Required for an Effective Oxidative Stress Response in Pseudomonas aeruginosa
Source: PLoS One. 2013 Jan 28;8(1):e54479. doi: 10.1371/journal.pone.0054479 (PMC3557286; doi:10.1371/journal.pone.0054479)
Supplement: Figure S5 — Comparative genomic analysis of the metabolic-centric PA2206 region. Analysis of all available P. aeruginosa genome sequences in which PA2206 homologues were identified, including P. fluorescens Pf-5 and P. fulva 12×, based on the Pseudomonas Genome Database. Single genes and operons are denoted by colour and pattern-fill. Gene content and organisation is highly conserved in P. aeruginosa strains. P. fluorescens encodes a PA2206 homologue, which is adjacent to truncated genes corresponding to fragments of PA2207 and PA2212, both of which are downstream of a conserved PA2214-2216 homologous operon. P. fulva encodes a TRAP system inserted between homologues of the PA2213 and PA2216 genes. (PPT) [file pone.0054479.s005.ppt]

## Slide 1
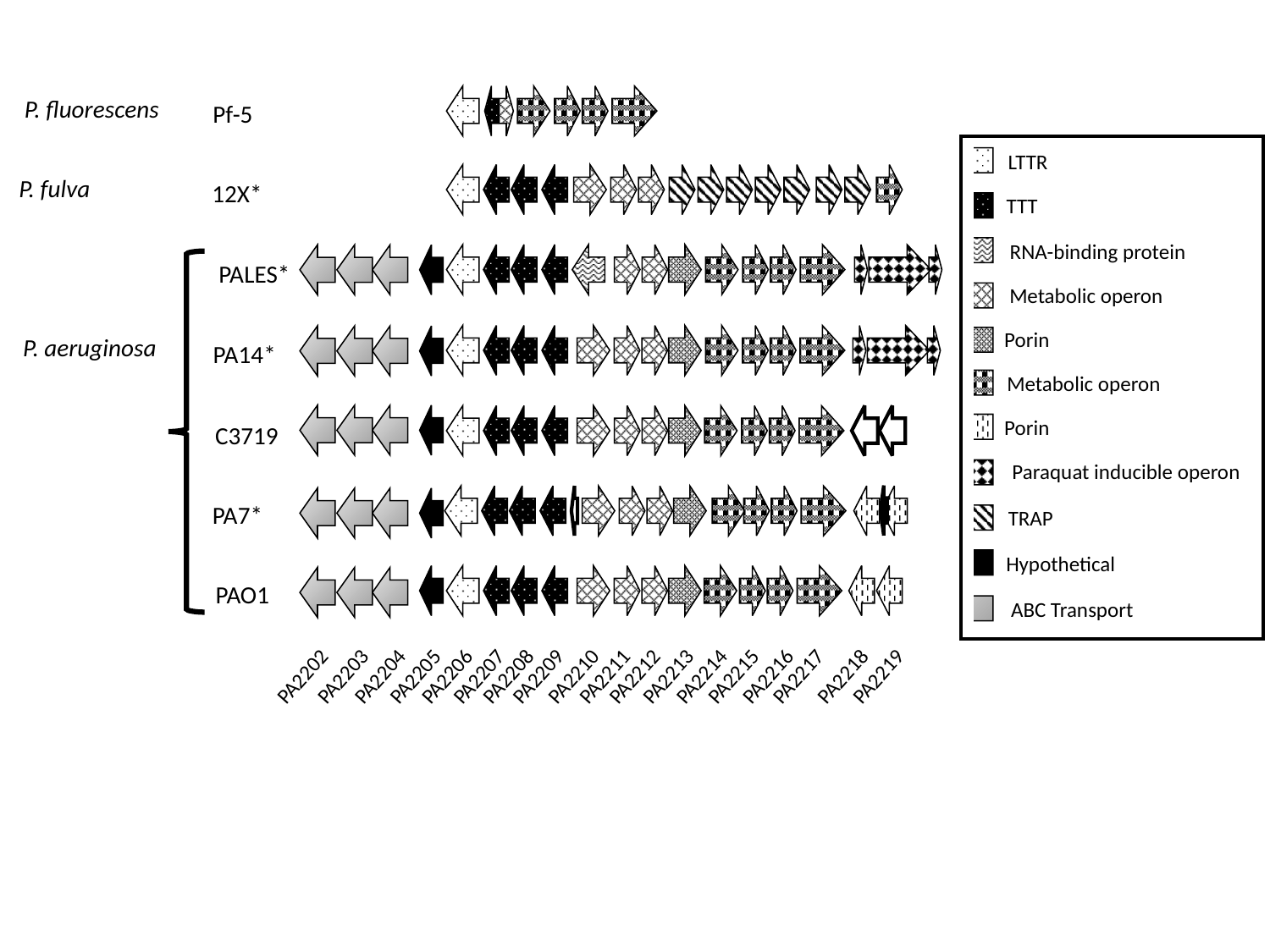

P. fluorescens
Pf-5
LTTR
P. fulva
12X*
TTT
RNA-binding protein
PALES*
Metabolic operon
Porin
P. aeruginosa
PA14*
Metabolic operon
Porin
C3719
Paraquat inducible operon
PA7*
TRAP
Hypothetical
PAO1
ABC Transport
PA2208
PA2209
PA2210
PA2215
PA2212
PA2217
PA2204
PA2205
PA2206
PA2213
PA2214
PA2219
PA2203
PA2211
PA2202
PA2216
PA2218
PA2207
