# Supplementary material for: A Non-Classical LysR-Type Transcriptional Regulator PA2206 Is Required for an Effective Oxidative Stress Response in Pseudomonas aeruginosa
Source: PLoS One. 2013 Jan 28;8(1):e54479. doi: 10.1371/journal.pone.0054479 (PMC3557286; doi:10.1371/journal.pone.0054479)
Supplement: Figure S6 — (A) Genome-wide BioPerl analysis of the 5′-TTGCCTGGGGTTA-3′ sequence revealed 18 promoters that contained putative LysR boxes with similarity (p<1e−04) to the PA2206 motif. MEME analysis of these LysR boxes led to the construction of a PA2206 consensus motif. Genes whose expression was altered in the PA2206C strain are highlighted in bold and the fold changes are indicated in parentheses. (B) EMSA analysis reveals a binding interaction between PA2206 and the PA4881 promoter, forming C1 and C2/C3 complexes in a concentration dependent manner. No interaction was observed at the PA1874 promoter, consistent with its lack of induction in the transcriptomic profile. (PPT) [file pone.0054479.s006.ppt]

## Slide 1
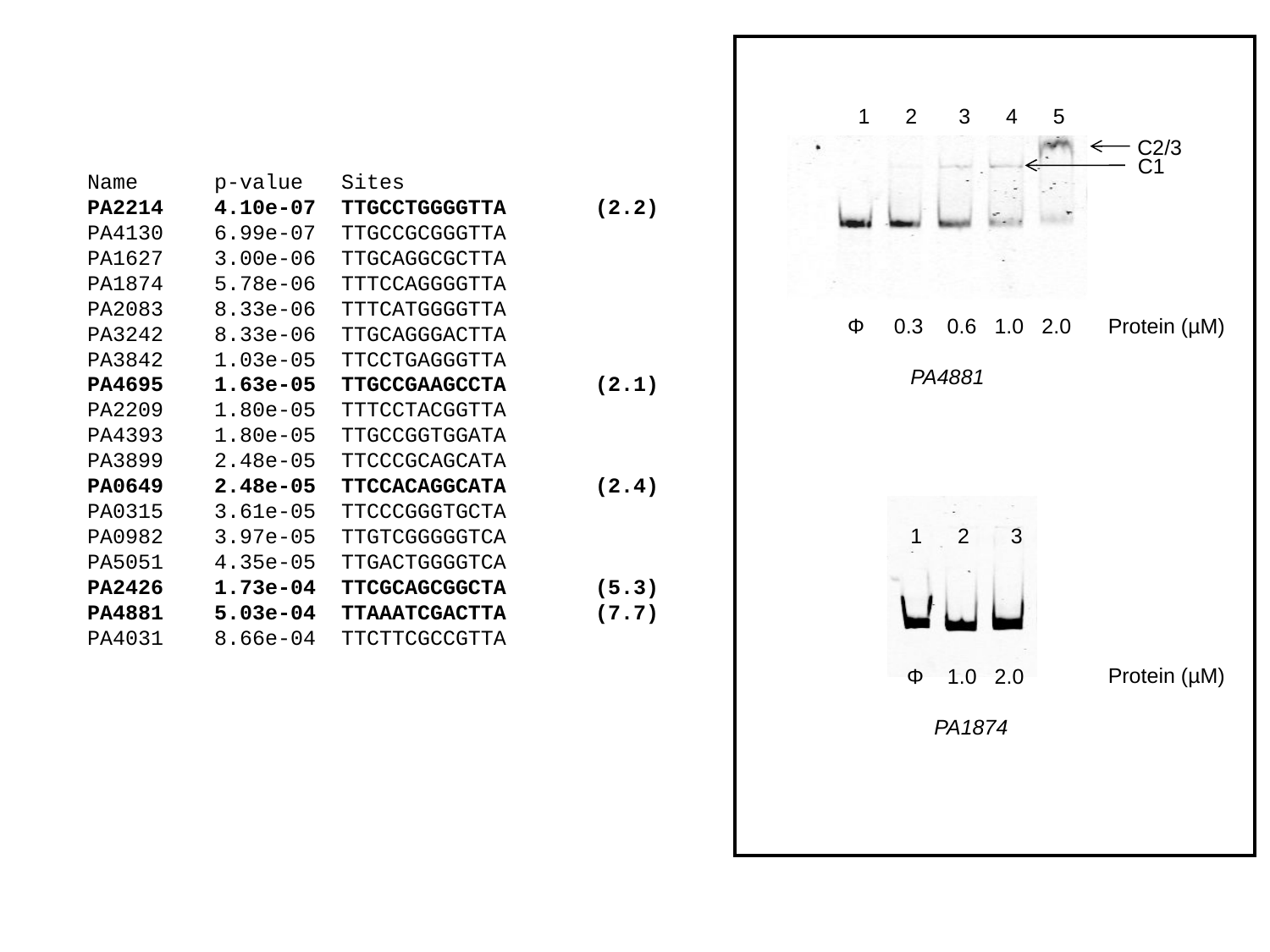

1 2 3 4 5
C2/3
Name	p-value	Sites
PA2214	4.10e-07	TTGCCTGGGGTTA	(2.2)
PA4130	6.99e-07	TTGCCGCGGGTTA
PA1627	3.00e-06	TTGCAGGCGCTTA
PA1874	5.78e-06	TTTCCAGGGGTTA
PA2083	8.33e-06	TTTCATGGGGTTA
PA3242	8.33e-06	TTGCAGGGACTTA
PA3842	1.03e-05	TTCCTGAGGGTTA
PA4695	1.63e-05	TTGCCGAAGCCTA	(2.1)
PA2209	1.80e-05	TTTCCTACGGTTA
PA4393	1.80e-05	TTGCCGGTGGATA
PA3899	2.48e-05	TTCCCGCAGCATA
PA0649	2.48e-05	TTCCACAGGCATA	(2.4)
PA0315	3.61e-05	TTCCCGGGTGCTA
PA0982	3.97e-05	TTGTCGGGGGTCA
PA5051	4.35e-05	TTGACTGGGGTCA
PA2426	1.73e-04	TTCGCAGCGGCTA	(5.3)
PA4881	5.03e-04	TTAAATCGACTTA	(7.7)
PA4031	8.66e-04	TTCTTCGCCGTTA
 Φ 0.3 0.6 1.0 2.0
PA4881
C1
Protein (µM)
1 2 3
Protein (µM)
 Φ 1.0 2.0
PA1874
