# Supplementary material for: A Non-Classical LysR-Type Transcriptional Regulator PA2206 Is Required for an Effective Oxidative Stress Response in Pseudomonas aeruginosa
Source: PLoS One. 2013 Jan 28;8(1):e54479. doi: 10.1371/journal.pone.0054479 (PMC3557286; doi:10.1371/journal.pone.0054479)
Supplement: Table S1 — Primers used in this study. (DOC) [file pone.0054479.s007.doc]

**Table S3. Primers used in this study.**

| Primer | Sequence 5’-3’ |
| --- | --- |
| *Protein Expression Primers* |  |
| 2206HisNR | CCCAAGCTTGGGTCACTTCGCCAGGTG |
| 2206HisF | CGGAATTCCGGAGATACGCCATGTCC |
| *Promoter Fusion Primers* |  |
| PA2206TRF | TCTAGATCAGCCTGATCGTGGTACTG |
| PA2206TRR | GGTACCCGGGACATGGCGTATCTC |
| PA2214TRF | GCTCTAGAGCCTGAAGAACCTCGGCCTG |
| PA2214TRR | GGGGTACCCATGGAGGGCTTCCTTC |
| PA2216TRF | GCTCTAGAGCGCCGAGTTCTTCCCGGTG |
| PA2216TRR | GGGGTACCCATCTCGGCTCCTCCTG |
| lacZ | GGGTAACGCCAGGGTTTTCC |
| *Quantitative RT-PCR Primers* |  |
| PA2206F | GAAGAACTCTGCGTGGTGGT |
| PA2206R | ATCATGTCGGTCTGTTGCAG |
| ProCF | GGCGTATTTCTTCCTGCTGA |
| ProCR | TGGCCTGGAAGGATTTGAT |
| PA5129F | CCTGGTGTCCGTACTGCAT |
| PA5129R | TCCAGTGCATGAAGGTCGT |
| PA0729.1F | gcgggcgtcgtataatgg |
| PA0729.1R | GGGCGAAGGGAATCGAAC |
| PA3032F | TTCCCAAGTTCCTCATCGAC |
| PA3032R | CCCGTTGAACAACTCGAAAT |
| PA2821F | GAAGAACGCTATCCCGAACC |
| PA2821R | GTCGAGATGGTTTTCCAGGT |
| *Complementation Primers* |  |
| PA2206CCF | GGAAGCTTCGCGCTCGGCTACCTGTTCAAG |
| PA2206CCR | GGTCTAGATCACTTCGCCAGGTGCCGCGC |
| Hin2206F | CCCAAGCTTGCCAGCGAGATACGCCATG |
| Xb2206R | CCCTCTAGAGCTCACTTCGCCAGGTGC |
| *InfraRed Promoter Primers* |  |
| PA2214F | CGATAACCGGCGATTGGC |
| PA2214F2 | CGCCTACGTAATCCAGAG |
| PA2214R | GCAGCCTGGCGTGGTCG |
| PA2214TrF | GGTGAAAAGGTATTGCC |
| PA2214TrR | GGCAATACCTTTTCACC |
| PA2214TrAF | CGATGAATGCGCAGAAG |
| PA2214TrAR | CATCAGGACCAGGAAAG |
| PA2214TrBF | ATGCGCAGAAGTGATTA |
| PA2214TrCF | GTGATTATCCGCGCGGG |
| PA2214TrDF | GGCCTGGCTACAGTCGG |
| PA2214TrEF | GCCATGCCGCCTCCGCG |
| PA2214TrER | CGGTGTCGGCTTGCAGG |
| PA2216F | GGTCAGTGCCGGTGATCC |
| PA2216R | CTGGATATAGCCGTCGAC |
| PA2206F | CGATGCTGTTCTCCGACG |
| PA2206R | GACATGGCGTATCTCGCT |
| PvdSF | GTCTGCGACGCATGA |
| PvdSR | GGCTGCAGAGCACTG |
| PvdSTrAF | GCTATTTGCCAGCATGC |
| PvdSTrR | TAATGCGGGCGGCGATC |
| PA1874F | GCCGTGCCAGGTGATCGG |
| PA1874R | CCGCACCATGTAATCCAG |
| PA4881EF | CGGCTTTTCTTTTCGCCTTA |
| PA4881ER | TGCGTTGGAGTCATTTTCAG |
| PA0982F | CTTCTTCAAGGCGACGC |
| PA0982R | CTCAGTCGCGGAAATCG |
