# Supplementary material for: A Non-Classical LysR-Type Transcriptional Regulator PA2206 Is Required for an Effective Oxidative Stress Response in Pseudomonas aeruginosa
Source: PLoS One. 2013 Jan 28;8(1):e54479. doi: 10.1371/journal.pone.0054479 (PMC3557286; doi:10.1371/journal.pone.0054479)
Supplement: Table S2 — Disk diffusion analysis of the oxidative stress sensitivity of wild-type P. aeruginosa and PA2206− and PA2215− mutants. Analysis performed using 10 µl of a 30% (w/w) solution of H2O2 (8.8 M). (DOC) [file pone.0054479.s008.doc]

**Table S2. Disk diffusion analysis of the oxidative stress sensitivity of wild-type *P. aeruginosa* and *PA2206^-^* and *PA2215^-^* mutants.**  Analysis performed using 10 µl of a 30% (w/w) solution of H_2_O_2_ (8.8 M).

**mPAO1 *PA2206^-^ PA2215^-^***

Average 22.5 26.4 27.5

St. Dev. 1.40 3.49 2.08

ttest 0.047 0.007
